# Adoptive Transfer of EBV Specific CD8^+^ T Cell Clones Can Transiently Control EBV Infection in Humanized Mice

**DOI:** 10.1371/journal.ppat.1004333

**Published:** 2014-08-28

**Authors:** Olga Antsiferova, Anne Müller, Patrick C. Rämer, Obinna Chijioke, Bithi Chatterjee, Ana Raykova, Raquel Planas, Mireia Sospedra, Anatoliy Shumilov, Ming-Han Tsai, Henri-Jacques Delecluse, Christian Münz

**Affiliations:** 1 Viral Immunobiology, Institute of Experimental Immunology, University of Zürich, Zürich, Switzerland; 2 Neuroimmunology and Multiple Sclerosis Research, Department of Neurology, University Hospital Zürich, Zürich, Switzerland; 3 Division of Pathogenesis of Virus Associated Tumors, German Cancer Research Centre (DKFZ), Heidelberg, Germany; University of Southern California Keck School of Medicine, United States of America

## Abstract

Epstein Barr virus (EBV) infection expands CD8^+^ T cells specific for lytic antigens to high frequencies during symptomatic primary infection, and maintains these at significant numbers during persistence. Despite this, the protective function of these lytic EBV antigen-specific cytotoxic CD8^+^ T cells remains unclear. Here we demonstrate that lytic EBV replication does not significantly contribute to virus-induced B cell proliferation *in vitro* and *in vivo* in a mouse model with reconstituted human immune system components (huNSG mice). However, we report a trend to reduction of EBV-induced lymphoproliferation outside of lymphoid organs upon diminished lytic replication. Moreover, we could demonstrate that CD8^+^ T cells against the lytic EBV antigen BMLF1 can eliminate lytically replicating EBV-transformed B cells from lymphoblastoid cell lines (LCLs) and *in vivo*, thereby transiently controlling high viremia after adoptive transfer into EBV infected huNSG mice. These findings suggest a protective function for lytic EBV antigen-specific CD8^+^ T cells against EBV infection and against virus-associated tumors in extra-lymphoid organs. These specificities should be explored for EBV-specific vaccine development.

## Author Summary

Epstein Barr virus persistently infects more than 90% of the human adult population. While fortunately carried as an asymptomatic chronic infection in most individuals, it causes B cell lymphomas and carcinomas in some patients. Symptomatic primary EBV infection, called infectious mononucleosis, predisposes for some of these malignancies and is characterized by massive expansions of cytotoxic T cells, which are mostly directed against lytic EBV antigens that are expressed during virus particle production. Therefore, we investigated the protective role of lytic EBV antigen specific T cells during EBV infection and the contribution of lytic EBV infection to virus-associated tumor formation. We found that lytic EBV antigen specific T cells kill B cells with lytic virus replication and might thereby transiently control EBV infection in mice with human immune system components. Furthermore, we observed that EBV associated B cell tumors outside secondary lymphoid organs may require lytic replication for efficient formation. Thus, we suggest that lytic EBV antigens should be explored for vaccination against symptomatic EBV infection and EBV associated extra-lymphoid tumors.

## Introduction

Epstein Barr virus (EBV) is a ubiquitous human γ-herpesvirus that establishes persistent infection in more than 90% of the human adult population [1]. Like other herpesviruses, EBV can undergo two infection programs. Latent infection leads to the expression of eight viral proteins and more than forty non-translated RNAs. This program is able to immortalize human B cells and is found in EBV associated tumors [2]. In contrast, lytic EBV infection leads to the expression of more than eighty lytic EBV proteins and the production of infectious viral particles [3]. Human T cells recognize both latent and lytic EBV antigens with distinct hierarchies [4]. While the latent nuclear antigen 1 (EBNA1) is a consistently recognized CD4^+^ T cell antigen [5], latent membrane protein 2 (LMP2) and the EBNA3 proteins are prominent CD8^+^ T cell antigens [6]. Furthermore, CD4^+^ T cells often recognize late lytic EBV antigens [7], while CD8^+^ T cells arise in response to early lytic antigens, including BMLF1 [8]. While EBV transformed B cells (Lymphoblastoid cell lines (LCLs)) can be readily recognized by CD8^+^ T cells specific for lytic EBV antigens, the protective value of lytic antigen-specific CD8^+^ T cells has not been readily demonstrated so far. Moreover, the expansion of these lytic EBV antigen-specific CD8^+^ T cells seems to follow viral load during symptomatic primary infection, called infectious mononucleosis, while latent EBV antigen-specific T cells peak during convalescence consistent with their involvement in viral immune control [4]. Thus, we aimed to evaluate the protective role of lytic EBV antigen specific CD8^+^ T cells *in vitro* and *in vivo*.

Due to its exclusive tropism for human cells, this oncogenic γ-herpesvirus is difficult to study *in vivo*. However, recent advances have led to the development of two informative *in vivo* models. One model examines the infection of rhesus macaques with lymphocryptoviruses (LCV), a subgroup of γ-herpesviruses that includes EBV [9], and the other model examines EBV infection in mice with reconstituted human immune system components [10]. In both systems, T cell targeted immunosuppression leads to loss of viral immune control and virus-associated tumor formation [11], [12], [13]. We have explored EBV infection of non-obese diabetic mice with a severe combined immunodeficiency mutation and with complete loss of the interleukin 2 receptor gamma chain locus (NOD-*scid IL2Rγ^null^* or NSG). These mice were reconstituted with human immune system components (huNSG mice). In this model, both CD4^+^ and CD8^+^ T cells contribute to adaptive immune control of EBV [13], [14]. Furthermore, it allows the assessment of innate immune responses by natural killer (NK) cells in response to EBV infection [14], [15] and the exploration of EBV-specific vaccine candidates targeted to dendritic cells [16], [17]. Finally, the infection of huNSG mice with EBV isolates and mutants with enhanced tumorigenesis replicate clinical features of EBV infection [18], [19]. Thus, this *in vivo* model of EBV infection recapitulates main features of EBV infection in humans and should allow us to interrogate the protective value of T cell responses against latent and lytic EBV antigens.

In this study, we demonstrated that wild-type (WT EBV) and BZLF1 deficient EBV (ZKO EBV), which lacks with BZLF1 one of the immediate early transactivators of lytic replication, replicate to similar viral titers in huNSG mice. However, BZLF1 deficient virus establishes B cell lymphomas less efficiently outside of secondary lymphoid tissues. Furthermore, CD8^+^ T cells specific for the lytic EBV antigen BMLF1 eliminate lytically EBV replicating B cells efficiently in LCL cultures *in vitro* and in huNSG mice *in vivo*, thereby transiently controlling EBV infection in huNSG mice. Thus, lytic EBV antigen-specific CD8^+^ T cells are able to target cells infected with lytically replicating virus. These antigens should be considered for EBV-specific vaccine formulations, and in particular for patients with uncontrolled primary lytic EBV replication such as infectious mononucleosis.

## Materials and Methods

### Mice

NOD-*scid IL2Rγ^null^* HLA-A2 transgenic (NSG-A2tg) mice were obtained from the Jackson Laboratory, and bred and raised under specific pathogen-free conditions at the Institute of Experimental Immunology, University of Zürich, Switzerland. Newborn NSG-A2tg mice (1 to 5 days old) were irradiated with 1 Gy and injected intrahepatically 5–7 hours later with 1–2×10^5^ HLA-A*02 positive CD34^+^ human hematopoietic progenitor cells. CD34^+^ cells were isolated as described previously from human fetal liver tissue (Advanced Bioscience Resources, Alameda, CA, USA) [13], [18]. The reconstitution of human immune system components in the peripheral blood of humanized NSG-A2tg mice (huNSG-A2tg) was analyzed for each cohort 12 weeks after engraftment and prior to the beginning of experiments.

### Ethics statement

All animal protocols were approved by the cantonal veterinary office of Zurich, Switzerland (protocol nos. 116/2008 and 148/2011). All studies involving human samples were reviewed and approved by the ethical committee of Zurich, Switzerland (protocol no KEK-St-Nr 19/08). These protocols follow the European Convention for the Protection of Vertebrate Animals used for Experimental and Other Scientific Purposes as well as the Swiss Animal Welfare Act (TSchG; 455) (Amendment of 15 June 2012) and the Swiss Animal Welfare Ordinance (TSchV; 455.1).

### Virus infection *in vitro*

Epstein-Barr virus B95-8 wild-type (WT EBV) and BZLF1 knock-out (ZKO EBV) virus were produced in 293 HEK cells (kindly provided by Regina Feederle and Henri Jacques Delecluse, Heidelberg, Germany). Titration of viral concentrates was performed on Raji cells using serial dilution and calculated as Raji infection units (RIU) using flow cytometric analysis of GFP-positive Raji cells 2 days after infection. Bulk peripheral blood mononuclear cells (PBMCs) were isolated from buffy coats by centrifugation using Ficoll-Paque, stained with carboxyfluorescein succinimidyl ester (CFSE) and incubated with mock, WT or ZKO EBV at a multiplicity of infection of 0.01 in the presence of cyclosporine A (CsA, 1 µg/ml). Cultures were harvested and counted every 2–3 days. Absolute numbers of cells were counted using trypan blue exclusion and assessed by flow cytometry. In order to maintain cultures in the logarithmic growth phase, they were maintained at a maximal density of 1×10^6^ cells/ml and supplemented with fresh media every 5 days. Assays were performed on PBMCs from two EBV-seropositive donors in duplicate.

### Infection of mice and adoptive transfer experiments

Fourteen to sixteen weeks after engraftment, huNSG-A2tg mice were injected with 10^5^ RIU of WT, ZKO EBV or with PBS intraperitoneally, bled weekly starting from week 2 and euthanized 6 weeks after infection. For adoptive transfer studies, mice received 1×10^6^ of LMP2- or BMLF1-specific CD8^+^ T cell clones intravenously one day prior to infection, and they were euthanized 4 or 6 weeks post-infection. Clones for adoptive transfer were selected based on their level of IFNγ secretion. All cell transfer experiments were performed with either of two independently obtained LMP2- or BMLF1-specific T cell clones. After termination of the experiments, body and spleen weight were determined. Abdominal organs were analyzed macroscopically for the presence of visible tumors.

### Immunohistochemistry

Tissue was fixed using 4% formalin and then paraffin embedded. For immunohistochemistry, anti-CD20 (L26, Cell Marque Lifescreen), anti-EBNA2 (PE2, Novocastra Laboratories Ltd), anti-NKp46 (BAF 2225, R&D Systems), anti-human CD68 (514H12, Novocastra Laboratories Ltd), anti-neutrophil elastase (NE, ab21595, Abcam), anti-gp350 (OT6, kindly provided by Dr. J.M. Middledorp, VU University Medical Center, The Netherlands) or anti-ZEBRA antibodies (AZ-69, Argene) were used and 4 µm sections were processed using standard procedures on a BOND-MAX automated immunohistochemistry system (Leica Microsystems). Isotype staining served as negative control and EBV-positive specimen of human tonsil or control or M81-infected huNSG-A2tg mouse tissue as positive controls. The absolute number of ZEBRA^+^ (BZLF1^+^, BZ.1^+^) cells per spleen section was counted in a blinded fashion and normalized to the total area of spleen section. The total area of spleen sections was determined using ImageJ Software. The number of CD20^+^, EBNA2^+^, NKp46^+^, CD68^+^ and NE^+^ cells was counted for 5 and 10 fields per spleen at ×200 and ×400 magnification, respectively.

### Quantification of EBV load by real-time PCR

Total DNA from the whole blood was extracted using NucliSENS (bioMérieux) according to manufacturer's instructions. Splenic tissue was processed using QIAmp tissue Kit (QIAGEN) using the manufacturer's protocol. Quantitative analysis of EBV DNA was performed by TaqMan (Applied Biosystems) real-time PCR technique as described [20] with modified primers for the *Bam*H1 W fragment (5′-CTTCTCAGTCCAGCGCGTTT-3′ and 5′-CAGTGGTCCCCCTCCCTAGA-3′) and a fluorogenic probe (5′-(FAM)-CGTAAGCCAGACAGCAGCCAATTGTCAG-(TAMRA)-3′). All PCRs were run on an ABI Prism 7700 Sequence Detector (Applied Biosystems) and samples were analyzed in duplicates. No EBV DNA was detected in the blood of mock-infected animals for the duration of the experiment. Mice were considered uninfected if EBV DNA was not detected in the blood and spleen during the experiment.

### Flow cytometry

To analyze B cell outgrowth in *in vitro* assays, cells were labeled using anti-CD19 (HIB19, Biolegend), anti-CD3 (UCHT1, Biolegend), and anti-CD23 (M-L233, BD Biosciences) antibodies. The composition of blood and spleen samples from humanized mice was analyzed using anti-human CD45 (HI30, Biolegend), anti-CD3 (MHCD1918, Invitrogen), anti-CD4 (RPA-T4, Biolegend), anti-CD8 (SK.1, Biolegend), and anti-CD19 (MHCD1917, Invitrogen) antibodies. Spleens were mechanically disrupted and filtered through a 70 µm cell strainer. Erythrocyte lysis in whole blood or in spleen suspensions was done using NH_4_Cl. Cell suspensions were stained with antibodies for 20 min at 4°C and washed. To sort EBV-specific CD8^+^ T cells, PBMCs were isolated using Ficoll-Paque, washed and stained using PE-labeled HLA-A*0201 dextramers complexed with the following peptides: HIV gag_77–85_ (SLYNTVATL), LMP2_426–434_ (CLGGLLTMV) and BMLF1_259–267_ (GLCTLVAML) (Immudex MHC Dextramer). For staining HLA-A*0201 dextramers loaded with the BRLF1_109–117_ (YVLDHLIVV) or with the LMP1_159–167_ (YLQQNWWTL) were used in addition. Cells were incubated with dextramers for 10 min at room temperature followed by antibody labeling with anti-CD3 (UCHT1, Biolegend), and anti-CD8 antibodies. To assess the purity of the clones, cells were stained using the antibody combination described in the assessment of the reconstitution above together with relevant dextramer or irrelevant dextramer as negative control. To assess the expression of homing and activation molecules, cells were labeled with anti-CD62L (DREG56, BD Pharmigen), anti-CCR7 (clone 150503, R&D Systems), anti-HLA-DR (L243, Biolegend) and anti-CD25 (M-A251 and 2A3, BD) antibodies. To detect cytotoxic granules in T cell clones, cells were stained as described, fixed, permeabilized and stained intracellularly using anti-perforin (δG9, BD Pharmigen) and anti-granzyme B (GB11, BD Pharmigen) with corresponding isotype controls. To detect lytically replicating B cells in killing assays, cells were labeled with anti-CD19 (HIB19, Biolegend) and anti-CD3 (MHCD0328, Invitrogen) antibodies, fixed, permeabilized and stained intracellularly using mouse anti-ZEBRA (BZ.1, Santa Cruz) primary antibodies (or isotype control) and secondary goat anti-mouse PE antibodies (BD Biosciences). Dead cells were excluded based on LIVE/DEAD fixable Aqua labeling (Invitrogen). Fixation and permeabilization steps prior to intracellular stainings were performed using BD Cytofix/Cytoperm Kit. Fluorescently labeled cell suspensions were analyzed on a BD FACS Canto II or BD LSR Fortessa flow cytometer (BD Biosciences). Flow cytometric data analysis was performed using FlowJo software.

### Generation of EBV-specific T cell clones

EBV-specific T cells were sorted from the blood of a healthy HLA-A*0201 positive EBV carrier using LMP2- or BMLF1-specific dextramers. The donor was genotyped as HLA-A*02, A*68, B*44, and B*07. Dextramer positive populations live CD3^+^CD8^+^ cells were single-cell sorted and plated onto irradiated PBMCs and LCLs as feeders in the presence of 1 µg/ml phytohemagglutinin L (PHA-L) and 150 U/ml hrIL-2 as previously described [21]. After 2 weeks, T cells were tested in a split well assay for IFNγ secretion upon re-stimulation with 1 µM of relevant peptides. T cells specifically recognizing the tested peptide were re-stimulated with the same protocol. They were tested on day 13 for phenotypic characteristics by flow cytometry and for functional T cell avidity in peptide titration assays.

### Sequencing of TRAV and TRBV gene rearrangements

The TRAV and TRBV chain repertoire was assessed using a primer set as previously described [22]. Primers were obtained from Biomers (Ulm, Germany). PCR amplification was performed in a 25 µl reaction volume containing Pfu polymerase Buffer, 200 mM deoxynucleotide triphosphate, 0.5 µM C3 primer, 0.5 U Pfu DNA Polymerase (all reagents were provided by Thermo Fisher Scientific, Reinach, Switzerland), 0.5 µM forward primer, and 100 ng cDNA. The cycling conditions were as follows: initial denaturation for 4 min at 95°C and 35 cycles of 95°C for 30 s, primer annealing at 60°C for 20 s, and primer extension at 72°C for 60 s, terminated by a final extension at 72°C for 10 min. The PCR product was validated by electrophoresis in a 2% agarose gel. Nucleotide sequencing of PCR products was performed at Microsynth (Balgach, Switzerland) with 30 pmol of reverse C1 primer. TCR gene designations follow the ImMunoGeneTics (IMGT) nomenclature (http://www.IMGT.org).

### T cell clone epitope avidity, MHC-restriction and degranulation assays

For peptide titration assays, 1×10^4^ cells of EBV-specific clones were incubated with synthetic cognate peptides (Shanghai Biochem) for 18 h. LMP2 peptides and BMLF1 peptides at 5 µM concentration served as an irrelevant control for BMLF1- and LMP2-specific CD8^+^ T cell clones, respectively. For co-culture assays, clones were incubated with HLA-A*02 mismatched or autologous lymphoblastoid cell lines (LCLs) in an effector to target (E:T) ratio of 1∶5. LCLs were optionally pulsed with 1 µM cognate peptide for 1 h at 37°C and washed extensively. Supernatants were used for IFNγ ELISA (Mabtech). For degranulation assays, clones were incubated with cognate or control peptide for 8 h and then stained intracellularly with perforin and granzyme B antibodies using the BD Cytofix/Cytoperm Kit.

### *In vitro* killing assay

To evaluate the cytotoxic activity of T cell clones against the autologous LCLs, we performed functional *in vitro* killing assays. Autologous LCLs were generated by incubating bulk PBMCs from the T cell clone donor with B95-8 supernatants and used as targets. AKBM cells, genotyped as A*24, A*31; B*35, B*51; C*09, and C*14, were used as a mismatched control target. EBV positive AKBM cells that express GFP upon switching from latent to lytic EBV replication were induced to enter lytic cycle by ligation with F(ab′)_2_ IgG and used directly for co-culture experiments [23]. AKBM or autologous LCLs were incubated with BMLF1- or LMP2-specific CD8^+^T cell clones for 18 h at an E:T ratio of 1∶1 and stained intracellularly using ZEBRA (BZ.1, Santa Cruz) antibodies. Specific lysis was determined by using the formula: % lysis = 100×(1−[(experimental ZEBRA^+^ with effectors − mean isotype ctrl)/(mean ZEBRA^+^ without effectors − mean isotype)]. Negative value for specific killing indicates an increase in number of ZEBRA^+^ B cells. Two to four replicates were used for each condition.

### HLA-genotyping

Genomic DNA was isolated from cell type of interest using Qiagen DNeasy Kit and genotyped by PCR-SSP method using HLA-A*/-B*/-DRB1* und HLA-DQB1* Kit (Ptotrans).

### Statistical analysis

All data were analyzed with the Mann Whitney test, unless otherwise stated. A p value of <0.05 was considered statistically significant. Statistical analysis and the generation of graphs was performed using Prism software (GraphPad Software).

### Accession numbers

The GenBank accession number for the complete B95-8 wild type sequence is AJ507799.2 (Table S3). The UniProtKB/Swiss-Prot identification of BMLF1-protein is Q04360. The UniProtKB/Swiss-Prot identification of LMP2-protein sequence is P13285. The Immune Epitope Database identification numbers of HLA-A*02-restricted BMLF1_259–267_ and LMP2_426–434_ epitopes are 20788 and 6568, respectively.

## Results

### Lytic replication does not significantly contribute to B cell transformation *in vitro*

**Figure 1:**
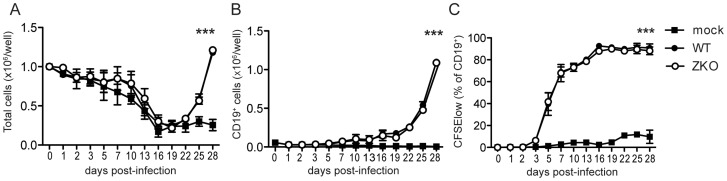
Lytic replication does not significantly contribute to EBV transformation *in vitro*. Bulk PBMCs were inoculated with WT EBV, ZKO EBV or mock treated in the presence of cyclosporine A. (**A**) Cells were harvested and absolute cell numbers per well were determined on indicated days. (**B**) Outgrowth of transformed CD19^+^ B cells was monitored using flow cytometry. (**C**) Proliferation of EBV transformed B cells was demonstrated by CFSE dilution and the subsequent increase in percentage of CFSE^low^ B cells. The experiments were performed in duplicate and data is presented as the mean ± SEM. Similar results were obtained in samples from two donors; the average of two experiments is presented (p _mock vs WT/ZKO_ <0.0001 by two-way ANOVA).

To understand the relevance of lytic infection and EBV lytic antigen-specific T cell immune responses in the control of EBV infection and virus-mediated B cell transformation, we used a recombinant EBV virus devoid of the lytic immediate early transactivator BZLF1 that is severely compromised to enter the lytic replication [24]. We first performed *in vitro* assays to assess if lytic replication itself has an effect on the initial transformation of B cells. Bulk peripheral blood mononuclear cells (PBMCs) from two EBV seropositive donors were infected with either WT or ZKO EBV with equal multiplicities of infection. This was done in the presence of cyclosporine A, an immunosuppressive drug that inhibits T cell responses, so that B cell outgrowth would not be affected by the presence of endogenous EBV-specific memory T cells in the assay. Three weeks post-infection, the absolute number of cells increased in the EBV-containing wells (Figure 1A), as previously reported for another EBV strain [25]. Flow cytometry analysis of the infected cells revealed that elevated cell numbers were due to the outgrowth of EBV transformed B cells (Figure 1B). Transformed B cells up-regulated expression of the cellular activation marker CD23 early after infection as compared to the non-infected control (Figure S1). Both viruses had similar initial transformation kinetics, which was confirmed by comparable CFSE dilution in both WT and ZKO EBV transformed B cells (Figure 1C, Figure S1). Additionally, these transformed cells revealed similar proliferation rates few months after transformation as reported previously (data not shown) [26]. Thus, lytic replication, as demonstrated using ZKO EBV, does not significantly contribute to the transformation of B cells and the subsequent proliferation of EBV-transformed B cell lines, referred to as lymphoblastoid cell lines (LCLs), *in vitro*.

### Effect of lytic replication on infection and extra-lymphoid tumorigenesis *in vivo*

**Figure 2:**
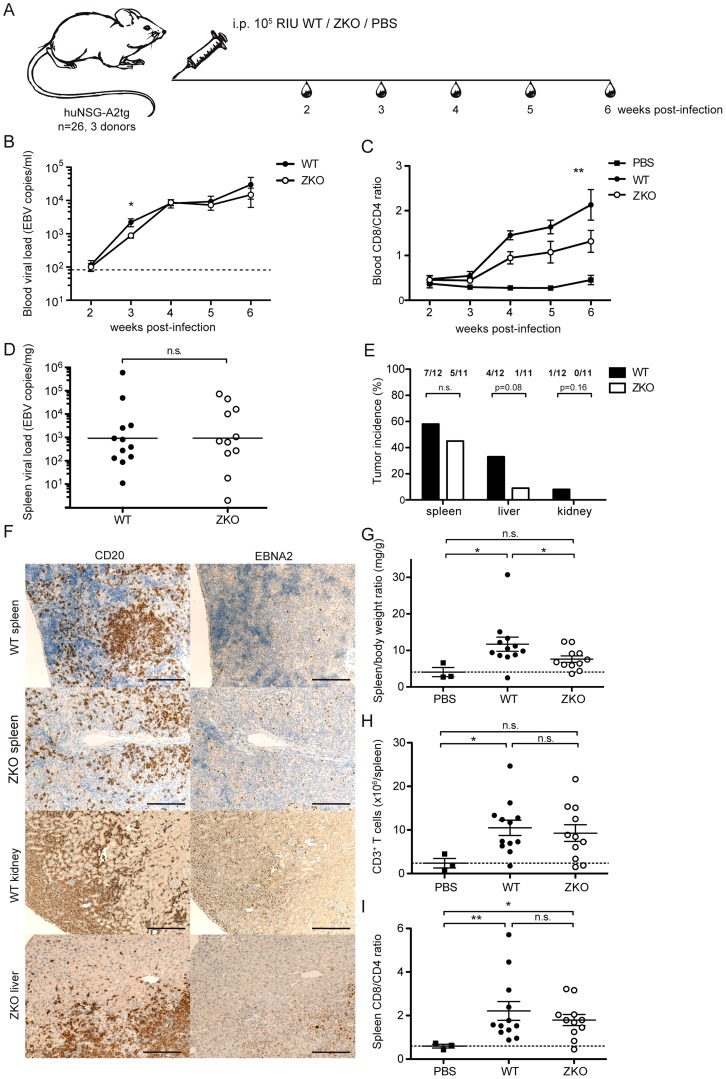
Lytic replication transiently influences viremia and may account to extra-lymphoid tumorigenesis in humanized mice. (**A**) Schematic diagram of *in vivo* infection experiments. Three cohorts of huNSG-A2tg mice were infected intraperitoneally (i.p.) with WT EBV, ZKO EBV or PBS, bled weekly starting from week 2 post-infection and euthanized at week 6. (**B**) Whole blood viral load at the indicated time points and presented as the mean ± SEM. Data for the initial four weeks represent composite data from 7 independent experimental cohorts of animals (n = 21–24 per group), data for the fifth and sixth represent composite data from 3 cohorts (n = 11–12 per group) (n.s. p _WT vs ZKO_ = 0.23 by two-way ANOVA; p _week 3: WT vs ZKO_<0.05 unpaired *t* test with Welch's correction). Dotted line represents detection limit. (**C**) Peripheral blood composition was analyzed using flow cytometry and the inversion of CD8-CD4 ratio was assessed weekly during the course of infection. Data is presented as the mean ± SEM (p _WT vs ZKO_ <0.001 by two-way ANOVA). (**D**) EBV DNA load was determined in spleen biopsies and presented as the geometric mean (n.s. p = 0.4 by unpaired *t* test). (**E**) Lymphoproliferative lesions of B cell origin were found in the organs of the abdominal cavity of some infected animals (spleen, liver and kidneys). Tumor burden is shown as percentage of tumor-bearing animals per group (n.s. p _WT vs ZKO_ = 0.09 by two-way ANOVA, n.s. p _liver:WT vs ZKO_ = 0.08 and p _kidney:WT vs ZKO_ = 0.16 by one-way Chi-square). (**F**) Immunohistochemical analysis of tumor sections confirmed the EBV-associated B cell origin of tumors by CD20 (**left panel**) and EBNA2 (**right panel**) staining. Scale bars, 200 µm. (**G**) Splenomegaly was assessed by comparing spleen weight to body weight. Data represents the mean ± SEM (p<0.05). (**H**) The absolute number of CD3^+^ T cells in the spleen was determined at the termination time point and represented as the mean ± SEM (p _PBS vs WT_ <0.05, n.s. p _PBS vs ZKO_ = 0.06, n.s. p _WT vs ZKO_ = 0.69). (**I**) The inversion of CD8:CD4 ratio in the spleen was determined by flow cytometry and represented as the mean ± SEM (n.s. p _WT vs ZKO_ = 0.98, p _PBS vs WT, PBS vs ZKO_ <0.05). Figures C–E represent composite data from three independent experiments with a total of 26 animals. Figure B represents composite data from seven independent experiments with a total of 45 animals.

To address whether lytic EBV replication has an effect on *in vivo* pathogenesis, we used a mouse model with reconstituted human immune system components (huNSG-A2tg mice). Three independent cohorts of humanized NSG-A2tg mice were infected with WT or ZKO EBV and monitored for 6 weeks after infection (Figure 2A). Whereas infrequent lytically replicating cells, which express BZLF1 protein (ZEBRA, BZ.1), were detected in the spleen sections of the majority of the WT EBV infected animals, no lytically replicating ZEBRA^+^ cells were detected in the spleen sections from ZKO EBV-infected mice (Figure S2). However, expression of the late lytic EBV antigen gp350 persisted in ZKO EBV-infected animals, which might indicate a low level of weak lytic replication in the absence of BZLF1 (Figure S2). EBV DNA was detected in the blood starting at week 2. The EBV virus titer rose rapidly until week 4 and moderately from week 4 to week 6 post-infection (Figure 2B). The detection of EBV DNA in whole blood in the first and second week post-infection, however, is limited by method sensitivity (<83 EBV copies/ml). WT and ZKO EBV replicated in the blood of infected animals with similar titers at all the time points, except for week three after infection. Combined data from seven independent cohorts of animals reveals a significantly lower viremia in the ZKO EBV infected animals at that time point (Figure 2B). Notably, while blood viremia in all animals infected with ZKO EBV rarely exceeded 3×10^3^ EBV copies/ml in the third week post-infection, half of the WT EBV infected animals reached up to 1×10^4^ EBV copies/ml in the blood (Figure 2B). This transient difference was, however, no longer observed at later time points. These findings suggest a low frequency of spontaneous EBV lytic reactivation, which transiently elevated viral loads in half of the WT EBV-infected humanized NSG-A2tg mice.

**Figure 3:**
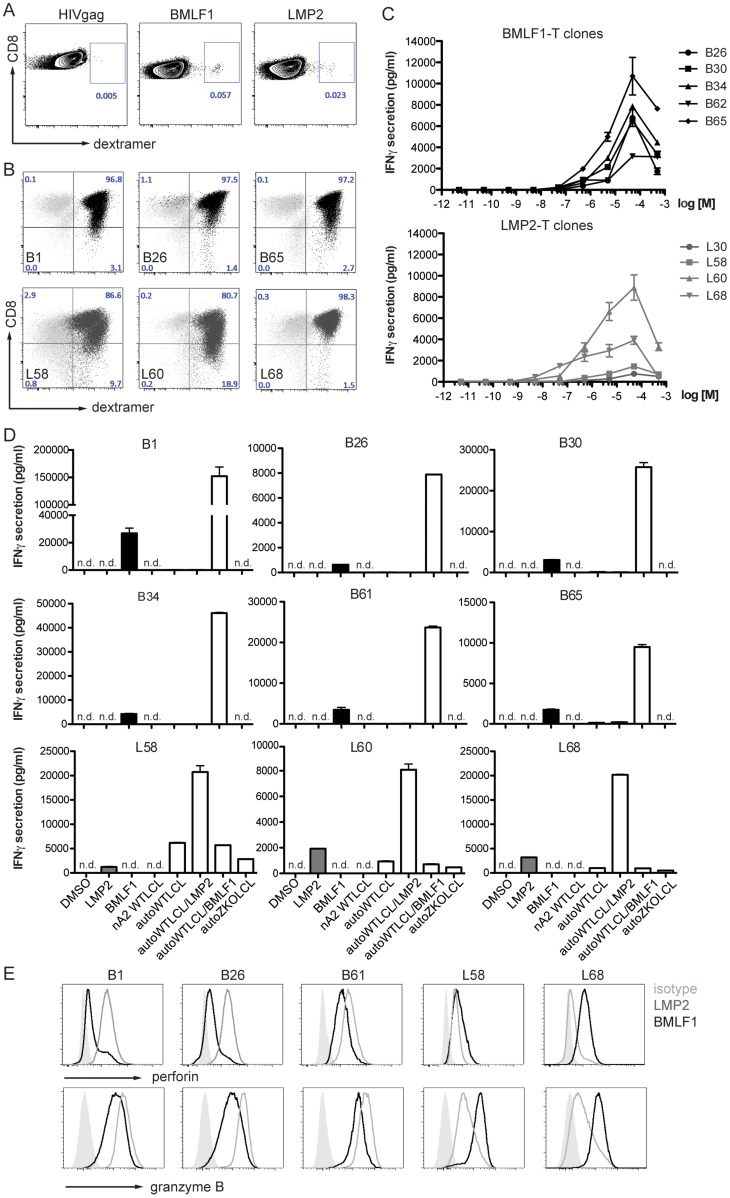
CD8^+^ T cell clones of lytic (BMLF1) and latent (LMP2) specificity were established by single cell sorting. (**A**) A dextramer positive population of live CD3^+^/CD8^+^ cells was sorted by flow cytometry from the blood of an HLA-A*0201 positive healthy EBV carrier. Flow cytometry plots show the frequency of starting populations within CD8^+^ T cells. (**B**) After two rounds of PHA re-expansions of sorted single cells, the purity of the T cell clones was confirmed by staining for T cell receptor specificity (MHC-dextramers), CD3, CD4, and CD8. Representative dextramer staining plots for three clones of LMP2-specificity (**dark grey**) and three clones of BMLF1-specificity (**black**) are shown, together with an overlay of control staining with an irrelevant dextramer (**light grey**). (**C**) Epitope avidity was determined in peptide titration assays for BMLF1-specifc clones (**top**) and LMP2-specific clones (**bottom**). The presence of IFNγ in supernatants upon stimulation with various concentrations of cognate peptide was assessed by ELISA and is presented as the mean ± SEM. Assays were performed in duplicate. (**D**) The ability to recognize HLA-A*02 negative WT LCLs, autologous WT LCLs and ZKO LCLs as well as peptide-pulsed WT LCLs was assessed for BMLF1-specific clones (**top panels**) and LMP2-specific clones (**bottom panel**) by IFNγ ELISA of supernatants after co-culture (n.d. – not detected). (**E**) FACS histograms of intracellular perforin and granzyme B staining for selected clones after stimulation with LMP2 (**grey**) and BMLF1 (**black**) peptides. An isotype control for intracellular staining is represented as a shaded grey histogram.

We observed an expansion of the CD8^+^ T cell compartment in the blood of infected animals, which is a hallmark of acute EBV-specific immune responses. Healthy EBV carriers as well as PBS-treated humanized mice maintain CD4^+^ cells as a majority of the CD3^+^ T cell population, whereas viral infection led to expansion of a subset of cytotoxic CD8^+^ T cells and the inversion of the CD8:CD4 ratio, which was more pronounced in WT animals compared to ZKO EBV infected animals (Figure 2C). Expanded T cells may be specific for the antigens expressed during the lytic phase of the EBV life cycle, which is reduced in ZKO EBV infected animals. While EBV-specific T cells could be detected by MHC dextramer staining in the blood of healthy EBV carriers (Figure 3A), the staining intensity for expanded blood and splenic CD8^+^ T cell populations in EBV-infected huNSG-A2tg mice was low (Figure S3), as previously reported [13], [27]. Interestingly, dextramer staining indicated also lytic EBV antigen specific T cell populations in the spleen of ZKO EBV infected animals, which along with the low level of gp350 staining in splenic sections could indicate a low level of weak lytic EBV replication in the absence of BZLF1. Despite this, the observed CD8^+^ T cell expansion suggests that adaptive cell-mediated responses are mounted against EBV antigens, similar to what has been observed in primary EBV infection.

Similar levels of EBV DNA were detected in the spleens of WT and ZKO EBV infected animals after six weeks of infection, while spleens of PBS-treated animals were free of EBV DNA (Figure 2D, data not shown). We also examined mice for the presence of EBV-associated lesions in the abdominal organs, such as spleen, liver and kidneys, 6 weeks after infection. As previously reported, ZKO EBV infection seemed to be less aggressive than WT EBV infection and leads to fewer lymphomas during six weeks post infection [28]. However, the composite data from four and six weeks experiments demonstrated no significant difference in total number of lymphomas between WT and ZKO EBV infected mice: 12 out of 24 WT EBV infected (50%) and 8 out of 21 ZKO EBV infected (38%) animals developed lymphomas in one or more abdominal organs. At least one macroscopically visible B cell tumor was observed in the spleen of 58% of WT EBV infected animals, while ZKO EBV caused splenic tumors in 45% of the infected animals 6 weeks post-infection. Despite this fairly similar frequency in tumorigenesis in the spleen, a detailed analysis of abdominal non-lymphoid organs revealed that ZKO EBV infected animals showed a trend to reduced numbers of lymphomas in the liver (p = 0.08, Figure 2E). A single mouse developed a kidney lymphoma six weeks after WT EBV infection. When combining four and six weeks experiments from 7 independent experimental cohorts with 21–24 animals per group the trend for loss of lymphomagenesis in the liver upon ZKO EBV infection was confirmed (p = 0.06). Histological analysis of the tumors confirmed the B cell origin of the lymphoproliferative lesions. This was concomitant with expression of virus-encoded nuclear antigen EBNA2, which is essential for the establishment of latent EBV infection (Figure 2F, Figure S4). Up to 60% of B cells of WT and ZKO EBV infected animals expressed EBNA2, revealing no difference between those viruses (Figure S4). Furthermore, the number of NK cells, macrophages and neutrophils were comparable in the spleens of WT and ZKO EBV infected animals (Figure S5). Macrophage and less often NK infiltration was a predominant feature of hepatic B cell lesions in WT EBV infected animals.

Most of the infected animals had splenomegaly, and this affected WT EBV infected animals more frequently (Figure 2G). The absolute number of B cells in the spleens remained unchanged upon infection with WT or ZKO EBV as compared to uninfected mice (data not shown). Splenomegaly in WT EBV infected animals resulted from the expansion of CD3^+^ T cells, while the expansion of T cells in the spleen was less pronounced in animals infected with ZKO EBV (Figure 2H). Despite moderate T cell expansion in the spleens of ZKO EBV infected animals, the CD8:CD4 ratio was inverted for most of the infected animals independent of lytic replication, indicating the enrichment of cytotoxic CD8^+^ T cells irrespective of lytic replication (Figure 2I).

These results indicate that lytic EBV replication affects viremia in the blood only transiently. Despite this, high viral loads lead to the subsequent expansion of CD8^+^ T cells. The increased occurrence of tumors in organs other than the spleen indicates that lytic replication might contribute to the establishment of lymphoproliferative disease outside of secondary lymphoid organs in huNSG-A2tg mice.

### Lytic but not latent EBV antigen-specific CD8^+^ T cells can eliminate lytically replicating cells

As we have previously reported, depletion of CD8^+^ T cells leads to poorly controlled WT EBV infection in the humanized NSG mouse model [13], [14]. In order to assess the role of EBV latent and lytic protein-specific CD8^+^ T cells in the control of EBV infection, we established numerous CD8^+^ T cell clones specific to representative immunodominant HLA-A*02 epitopes such as LMP2_426–434_ (latent) and BMLF1_259–267_ (lytic) from the blood of an HLA-A*0201 positive healthy EBV carrier (Figure 3A). After two rounds of re-expansion using the unspecific T cell mitogen phytohemagglutinin plus interleukin 2 *in vitro*, the cells were confirmed by dextramer straining to be pure clonal CD8^+^ T cell populations. Residual activation-driven CD8 down-regulation was observed for some clones early after re-stimulation (Figure 3B). For further characterization we sequenced T cell receptor variable genes of obtained several clones (Table S1). We observed frequent TRBV20-1 usage by BMLF1-specific CD8^+^ T cell. Whereas, each of BMLF1-specific CD8^+^ T cell clones had a unique CDR3β and CDR3α sequence, two out of three LMP2-specific clones shared the same CDR3β and CDR3α sequences, indicating their common clonal origin and explaining their similar behavior *in vitro*. We also observed frequent TRVB5-1 usage by LMP2-specific T cell clones. In peptide titration assays, the functional avidity for LMP2-specific clones was greater than for BMLF1-specific clones (13.8±3.2×10^−7^ M and 4.4±2.7×10^−6^ M, respectively, Figure 3C, Table S2).

To assess if these CD8^+^ T clones were able to recognize cognate antigen in the context of MHC class I molecules, we performed co-culture experiments with autologous or allogenic HLA-A*02 negative EBV-transformed LCLs. BMLF1-specific clones secreted limited amounts (less than 260 pg/ml) of IFNγ upon co-culture with autologous WT EBV-transformed LCLs (WT LCLs). In contrast, no IFNγ was detected upon co-culture with control HLA-A*02 negative WT LCLs (nA2 WT LCLs) or LCLs deficient in lytic replication (ZKO LCLs) (Figure 3D, upper panels). LMP2-specific CD8^+^ T cell clones were capable of recognizing both autologous LCLs (WT LCLs and ZKO LCLs) expressing endogenously processed LMP2 protein, indicating sufficient presentation of LMP2 by cell lines. Interestingly, in two independent experiments the amount of IFNγ secreted by three LMP2-specific T cell clones in response to autologous ZKO LCLs accounted for 49±2% and 20±10% of IFNγ secreted towards WT LCLs (Figure 3D, lower panel). As expected, recognition of autologous WT LCLs by either BMLF1- or LMP2-specific clones was improved dramatically when targets were pre-incubated with cognate but not irrelevant peptide (Figure 3D).

In order to address whether the BMLF1- and LMP2-specific clones were cytotoxic, we first assessed the presence of the pore-forming protein perforin and the cell death inducing serine protease granzyme B by intracellular staining. While incubation with irrelevant peptide resulted in high levels of perforin and granzyme B in these clones, stimulation with relevant peptide decreased the intensity of the staining, indicating the release of cytotoxic granules upon binding of the TCR with the cognate antigen (Figure 3E).

**Figure 4:**
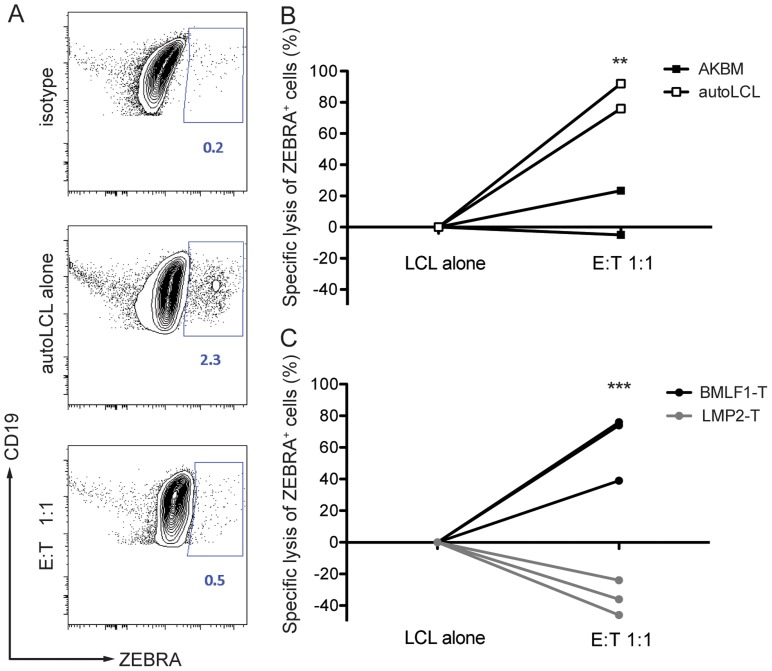
CD8^+^ BMLF1-specific clones eliminate lytically replicating EBV transformed B cells *in vitro*. Selected BMLF1-CD8^+^ T cell clones were co-cultured with lytically replicating EBV transformed B cells as targets overnight at an E:T ratio of 1∶1. (**A**) The percentage of lytically active LCLs was determined using intracellular ZEBRA staining by flow cytometry. Representative FACS plots demonstrate the isotype control background (**top**), population of steady state autologous ZEBRA^+^ LCLs when cultured alone (**middle**) and population of autologous ZEBRA^+^ LCLs remaining after co-culture with BMLF1-specific clonal CD8^+^ T cells (**bottom**). Percentages of ZEBRA^+^ cells were determined within CD19^+^ B cell populations pre-gated on live CD3^neg^ cells. (**B**) Specific lysis of autologous ZEBRA^+^ LCLs compared to lysis of mismatched ZEBRA^+^ AKBM is summarized for two independent experiments. A minimum of two biological and technical replicates were used for each of the experiments (p _LCL alone vs E:T 1∶1_<0.05, p _AKBM vs autoLCL_ <0.005 by two-way ANOVA). (**C**) Specific lysis of autologous ZEBRA^+^ LCLs by BMLF1-specific clones compared to lysis by LMP2-specific CD8^+^ T cell clones is summarized for three independent experiments. The experiments were performed with two biological replicates and at least two technical replicates (p _LCL alone vs E:T 1∶1, BMLF1-T vs LMP2-T_ <0.001 by two-way ANOVA).

To demonstrate that BMLF1-specific CD8^+^ T cell clones were able to eliminate autologous lytically EBV replicating LCLs in an MHC class I restricted manner, we co-cultured BMLF1-specific clones with autologous LCLs that spontaneously enter lytic replication (B95-8 EBV strain), or with HLA-mismatched AKBM cells that require the induction of lytic replication (Akata EBV strain) at a 1∶1 effector:target ratio. After overnight co-culture, the presence of lytically active LCLs was assessed using intracellular BZLF1 (ZEBRA, Zta, BZ.1) staining. The rate of spontaneous lytic replication in autologous LCLs was very low and reached a maximum of 2.3% of total B cells at high-density growth conditions (Figure 4A), while induced lytically active AKBM cells accounted for 30% of total B cells in culture (data not shown). Several independent BMLF1-specific CD8^+^ T clones efficiently eliminated ZEBRA^+^ cells from autologous co-cultures (74–90% of ZEBRA^+^ LCLs), while unspecific killing of mismatched AKBMs did not exceed 24% (Figure 4B). As a control, co-cultures of autologous LCLs with LMP2-specific clones led to increased frequencies of ZEBRA^+^ cells as compared to control wells without T cells, which is likely due the killing of latently infected B cells and the subsequent increase in the relative number of ZEBRA^+^ cells (Figure 4C). Thus, BMLF1-specific CD8^+^ T cell clones can eliminate LCLs with lytic EBV replication *in vitro* and might restrict the horizontal transmission of EBV through release of newly synthesized viral infectious particles.

### BMLF1-specific clones can transiently control EBV infection *in vivo*

**Figure 5:**
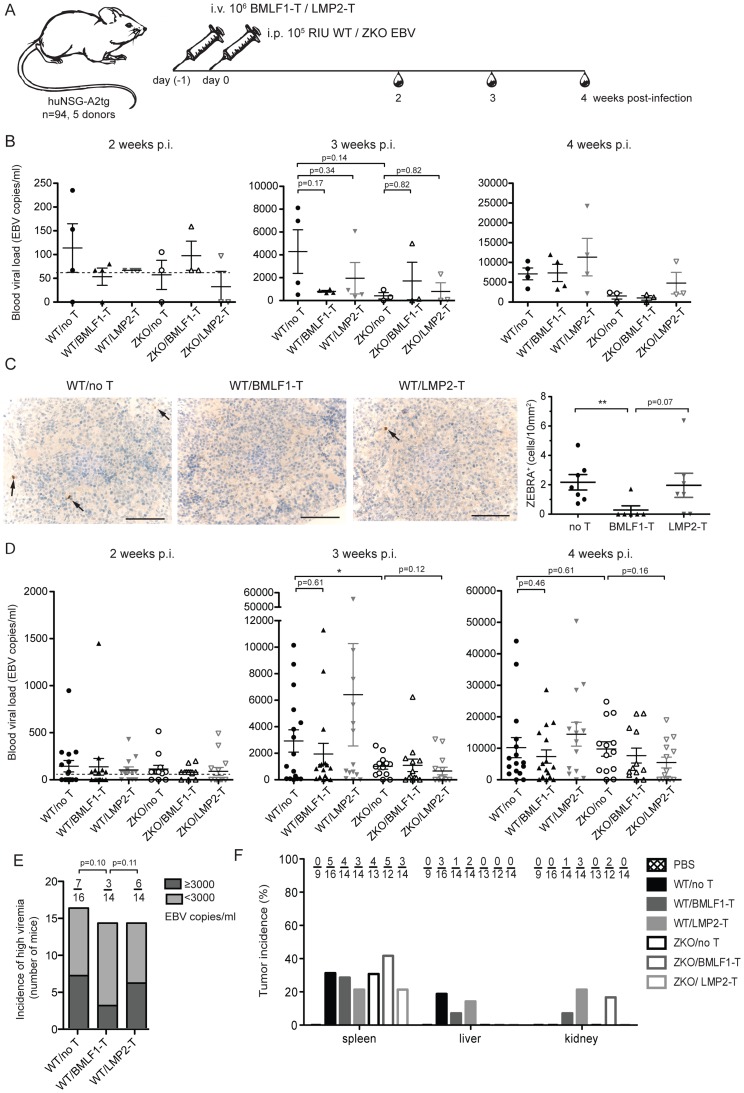
Lytic EBV antigen specific CD8^+^ T cell clones can transiently control EBV infection in humanized mice. (**A**) Schematic diagram of adoptive transfer experiments. BMLF1- or LMP2-specific CD8^+^ T cell clones were transferred i.v. into humanized NSG-A2tg mice. The next day, mice were infected i.p. with WT EBV, ZKO EBV or PBS. Mice were bled weekly starting at week 2 post-infection and were euthanized at week 4 or 6 post-infection (n = 94). (**B**) Whole blood EBV loads for the second, third and fourth week post-infection of mice, which received BMLF1- or LMP2-specific CD8^+^ T cell clones prior to WT or ZKO EBV infection, is demonstrated for a representative experiment (cohort 2, n = 3–4 per group, total n = 21). Dotted line represents detection limit. (**C**) Representative figures and quantification of the ZEBRA^+^ cells in the spleen sections of WT EBV infected huNSG-A2tg mice, which received no T cells, BMLF1-specific T cell clones and LMP2-specific T cell clones for two initial experiments (n = 6–7 per group, total n = 20). Data represent the mean ± SEM (p _no T vs BMLF1-T_ <0.01, p _BMLF1-T vs LMP2-T_ = 0.07). Scale bars, 100 µm. (**D**) Whole blood EBV loads for the second, third and fourth week post-infection of mice from five independent adoptive transfer experiments (n = 94). Data are represented as the mean ± SEM. (**E**) Incidence of high blood viremia on the third week post-infection in the WT EBV infected mice, which received no T cells, BMLF1-specific T cell clone and LMP2-specific T cell clone (n.s. by one-way Chi-square). (**F**) Tumor incidence per organ is presented as the percentage of animals which developed visible tumors in the organs of the abdominal cavity (n = 92).

In order to assess if BMLF1- and LMP2-specific CD8^+^ T cell clones were able to ameliorate EBV induced pathogenesis *in vivo*, we performed adoptive transfer experiments in huNSG-A2tg mice. Five independent cohorts of humanized NSG-A2tg mice were injected intravenously with BMLF1- or LMP2-specific CD8^+^ T cells in order to mimic endogenous circulating effector T cell populations, which could for example be induced by vaccination and indeed the transferred clones carried an effector memory phenotype after expansion (Figure S6). On the next day, mice were infected with either WT or ZKO EBV and the development of lymphoproliferative disease was monitored over 4 or 6 weeks (Figure 5A, Table S2). Adoptive transfer of a BMLF1-specific CD8^+^ T cell clones tended to reduce blood viremia in WT EBV infected animals 3 weeks post-infection, a time-point when EBV lytic replication results in elevated blood viremia in humanized NSG-A2tg mice (Figure 2B and 5B). Furthermore, ZEBRA^+^ B cells, containing lytically replicating WT EBV, were diminished in splenic sections of the majority of WT EBV infected animals upon transfer of BMLF-1 specific CD8^+^ T cell clones (Figure 5C). No ZEBRA^+^ cells were found in splenic sections from ZKO EBV infected animals (Figure S2). However, the amelioration of the EBV viremia in the blood of WT EBV infected animals by adoptive transfer of BMLF1-specific CD8^+^ T cell clones varied in the experimental cohorts of humanized NSG-A2tg mice (Figure 5D). Taken together, these five adoptive transfer experiments demonstrated that animals which received BMLF1-specific CD8^+^ T cells prior to WT EBV infection were less likely to develop high blood viremia (defined as greater than 3×10^3^ EBV/ml) at three weeks post WT EBV infection: only 3 animals out of 14 (21%) developed high viremia after BMLF1-specific T cell transfer as compared to 6 out of 14 (43%) after LMP2-specific T cell transfer or 7 out of 16 (44%) animals in the non-treated group (Figure 5E). Adoptive transfer of LMP2-specific T cells did not seem to have an ameliorating effect in WT EBV infected animals, however ZKO EBV infected animals might have benefitted from the T cell infusion on the third and fourth week after infection, possibly due to elimination of latently infected B cells from blood circulation.

Adoptive transfer of partially mismatched T cells in humans is considered relatively safe [29], despite rare cases of mild graft-versus-host disease [30]. Humanized mice, which received partly matched cytotoxic T cell clones, had no signs of graft-versus-host disease during the experiment. We attempted to identify the defining characteristics for protective EBV specific T cell clones by examining peptide epitope avidity, functional potency and surface phenotype, but we were unable to identify any *in vitro* predictors for *in vivo* protection. Additionally, attempts to find the transferred cells by specific amplification of mismatched MHC class I alleles of the donor using DNA from the blood at weeks 2, 3 and 4 post-infection, as well as in spleens and tumor samples upon termination of the experiments were limited by the sensitivity of our PCR-based method (1% of CD8^+^ T cells) and did not detect persistence of the transferred clonal T cell populations (data not shown).

We also assessed the effect of adoptively transferring lytic and latent antigen specific T cell clones on the formation of lymphoproliferative disease induced by WT and ZKO EBV. Consistent with our earlier experiments, EBV infected animals developed splenic EBV-associated B cell lymphomas with comparable frequency irrespective of lytic replication, whereas extra-lymphoid lymphomas were less frequent when EBV lytic replication was impaired (Figure 5F). Lymphomas from both WT and ZKO EBV infected animals contained EBNA2^+^ B cells, indicating the latency III program of the EBV life cycle. This form of latency is characterized by expression of all latent EBV antigens, namely EBNA proteins 1, 2, 3A, B, C, -LP, and LMP proteins 1 and 2. Latency III is commonly found in the lymphoproliferative lesions of immune-compromised patients.

Our study demonstrates subtle effects of EBV lytic replication on blood viremia and suggests that endogenous EBV lytic antigen-specific T cells might be primed in the huNSG-A2tg mice. Furthermore, we observed that EBV lytic replication may be required for the establishment of extra-lymphoid lymphoproliferative lesions in our mouse model. In healthy EBV carriers, memory CD8^+^ T cell specific for lytic and latent antigens of EBV are found in significant numbers. In this study, we provide the first evidence that BMLF1-specific CD8^+^ T cells eliminate EBV-transformed B cells undergoing lytic replication *in vitro* and *in vivo*, and are able to transiently control EBV viremia in the blood of infected humanized mice.

## Discussion

One form of uncontrolled lytic EBV replication is infectious mononucleosis. This symptomatic primary EBV infection occurs more frequently when primary viral infection is delayed into adolescence. It is characterized by high viral titers, particularly in saliva, and by CD8^+^ T cell lymphocytosis [31]. Otherwise, pathology by lytic replication of EBV is rare in healthy human carriers and EBV transformed lymphoblastoid cell lines switch only at low frequencies into lytic replication *in vitro*. Despite these findings, the role of lytic virus replication during EBV infection is only poorly understood. This is in part because the number of experimental systems that are permissive for EBV infection limits studies on EBV biology *in vivo*. We therefore examined EBV lytic replication in humanized NSG-A2tg mice and the functional role of BMLF1-specific T cells in the control of EBV infection *in vivo* and *in vitro*. This in vivo model of EBV infection recapitulates hallmarks of innate and adaptive cell-mediated immune control of EBV, but lacks the development of humoral immune responses and efficient mucosal homing of human immune cells [10].

Our longitudinal *in vivo* study suggests that EBV lytic replication is limited in our humanized mouse model and plays a transient role in dissemination of the EBV early after infection and prior to innate and adaptive immune control of lytically EBV replicating cells: only mice infected with lytic replication competent EBV, but not lytic replication compromised EBV, developed high viremia in the blood on the third week post-infection.

The massively expanding CD8^+^ T cells in infectious mononucleosis patients are mostly directed against lytic EBV antigens [32], suggesting that primarily lytic viral replication is ill-controlled during infectious mononucleosis. Particularly, CD8^+^ T cells that are specific for immediate early and early EBV antigens are observed at high frequencies [33]. These events correlate with more efficient LCL recognition by early lytic EBV antigen specific CD8^+^ T cells, while late lytic EBV antigen recognition by specific CD8^+^ T cells is presumably compromised by viral immunoevasins targeting MHC class I antigen presentation [34]. In our humanized mouse model we observed that high blood viral load frequently correlated with the number of EBV-specific T cells found in the spleens of the WT and ZKO EBV infected animals as detected by IFNγ ELISPOT assays (data not shown). Also, we report the inversion of the CD8:CD4 ratio in blood of infected animals, which is less pronounced in animals infected with EBV that is inhibited in its lytic replication. Our lab previously demonstrated that a significant proportion of EBV-specific T cells are primed specifically to lytic antigens rather than latent antigens in humanized NSG-A2tg mice [13]. These data suggest that lytic EBV antigens contribute to CD8^+^ T cell expansion after infection.

However, in our current study, we observe more EBV-specific T cells among splenocytes from both WT and ZKO EBV infected mice, if we stimulated the splenocytes with LCLs capable of lytic EBV replication (data not shown). Together with the observation *in vitro* that LMP2-specific CD8^+^ T cell clones consistently secreted more IFNγ in response to autologous WT LCL than to ZKO LCL stimulation, this might indicate decreased antigen presentation of LCLs deficient in lytic replication. Additionally, detection of EBV-specific T cells using HLA-A*02 dextramers bound to human immunodominant epitopes of EBV lytic and latent proteins was difficult due to low staining intensity (Figure S3). The process of T cell education of the T cells in humanized mice remains unclear. In these mice human T cells are educated on mouse thymic epithelial cells, human bone marrow derived cells or transgenic human MHC class I molecules stabilized by murine β2-microglobulin. We can speculate that this may result in T cells with TCRs, that are suboptimally recognized by human HLA-A*02 dextramers. Alternatively, maintenance of high TCR density on T cells in the mouse host might be compromised by lack of human cytokines and further compromised due to the high antigenic load after EBV infection.

Pudney and colleagues demonstrated direct recognition of lytically infected cells by CD8^+^ specific cells obtained from infectious mononucleosis patients [34]. However, in these studies, the authors could only demonstrate cytokine production by lytic EBV antigen specific CD8^+^ T cell clones in response to wild-type, but not BZLF1-deficient LCLs. Extending these findings, we can now document that BMLF1 specific CD8^+^ T cell clones eliminate ZEBRA expressing EBV infected B cells in LCL cultures. BMLF1-specific clones eliminated EBV lytically replicating autologous LCLs, but not MHC-class I mismatched control LCLs. Thus, lytic EBV antigen specific T cell clones contribute to the immune control of EBV infection by eliminating lytically virus replicating cells.

Another EBV-associated pathological condition in solid-organ and hematopoietic stem cell transplant patients is post-transplant lymphoproliferative disease (PTLD). This disease almost exclusively involves EBV-associated B cell proliferations characterized by expression of all latent proteins of EBV (latency III). In immune-compromised PTLD patients, mostly polyclonal B cell lesions could be found in secondary lymphoid organs and extra-lymphoid tissues [2]. EBV infection of humanized mice leads to the formation of lymphoproliferative B cell lesions resembling PTLD in histology and expression pattern of viral proteins. EBER^+^ EBV-transformed B cells were found in similar multifocal sites independently of lytic replication in humanized NSG mice, which were implanted with human fetal thymus and liver fragments in addition to the fetal liver derived CD34^+^ stem cell transfer of our model [28]. Our study for the first time demonstrates that EBV may require lytic replication to efficiently establish lymphomas in non-lymphoid tissues like kidneys and liver. This could result from decreased expression of growth factors in ZKO-derived LCLs, which may be more critical for the establishment of lymphoproliferative lesions outside of secondary lymphoid organs [26].

One therapy for patients with PTLD is adoptive T cell transfer. Both CD8^+^ and CD4^+^ EBV-specific T cells are being explored for adoptive transfer into PTLD patients [35], [36], [37], [38], [39]. Here we report, that adoptive transfer of BMLF1-specific CD8^+^ T cell clones into WT EBV infected humanized NSG mice transiently controlled EBV infection and was able to eliminate BZLF1 expressing cells from huNSG-A2tg spleens. However, given the limitation of detecting lytically replicating cells indirectly by examining blood viremia, this transfer did not result in significant and reproducible long-term control of EBV infection. We addressed whether fluctuations in the control of lytic replication *in vivo* was due to the characteristics of the transferred clones (epitope avidity, phenotype etc). However, no clear correlations could be found (Table S2). Alternatively, the inefficiency of clonal T cell transfer may result from the overall HLA mismatch – except matched HLA-A2 expression – between reconstituted human immune system components and transferred T cell clones. This might have led to their alloreactive rejection by the host and the limited persistence of the transferred cells *in vivo*. Clinical studies suggest that a positive outcome of cytotoxic T cell transfer into PTLD patients positively correlates with the matching of donor T cells to the recipient [38]. Despite this, an ameliorating effect of adoptive transfer in PTLD patients could also be observed in partly matched settings [30], [39]. Within five cohorts of huNSG-A2tg mice reconstituted with one (Cohorts 2, 3) or more (Cohorts 1, 4, 5) matched HLA alleles, the most pronounced effect of BMLF1-specific clone on viremia in blood was observed irrespective of the number of matched MHC class I alleles (Cohorts 1 and 2 at week 3, cohort 3 at week 4). Likewise, adoptive transfer of partly matched T cell clones in primates revealed no correlation between persistence of the infused cells and genetic backgrounds of donor and recipient [40]. Taken into account that WT EBV infection caused high viremia in half of the infected humanized NSG-A2tg mice, our study indicates that BMLF1-specific CD8^+^ T cells seem to maintain some immune control and can eliminate B cells undergoing lytic replication. Our data provide a rationale for reconsidering lytic EBV antigens for EBV specific vaccination, particularly during uncontrolled lytic replication and to limit EBV associated tumorigenesis in non-lymphoid tissues, which seems to be supported by lytic replication *per se.* Accordingly, lytic EBV antigens should be explored as vaccine components to mitigate infectious mononucleosis.

Currently, several EBV specific vaccine candidates are being explored. These include recombinant gp350 protein formulations that aim to elicit neutralizing antibodies against the main attachment protein for complement receptor 1 and 2 (CD35 and CD21) dependent B cell infection by EBV [41], [42], [43]. Furthermore, recombinant viral vaccines encoding latent EBV antigens, for example modified vaccinia virus Ankara (MVA), encoding a EBNA1-LMP2 fusion protein, are being explored [44], [45]. EBV derived virus like particles devoid of viral DNA have been investigated as vaccine candidates [46], [47], [48], [49]. Finally, we have been characterizing EBNA1 targeting to dendritic cells as a vaccine formulation [16], [17], [50]. However, all of these vaccination schemes include either latent or late lytic EBV antigens. They do not provide any stimulation for the immediate early and early lytic EBV antigen specific CD8^+^ T cells, which constitute the most frequent EBV specificities in both symptomatic primary infection and healthy EBV carriers. Since these specificities, nevertheless, efficiently recognize and eliminate lytically EBV replicating B cells ([34] and our data), they should be included into vaccine formulations against infectious mononucleosis and possibly also against EBV associated malignancies in extra-lymphoid tissues.

## Supporting Information

Figure S1 **ZKO EBV deficient in lytic replication leads to up-regulation of B cell activation marker CD23 and subsequent proliferation.** Bulk PBMCs were inoculated with WT EBV, ZKO EBV or mock treated in the presence of cyclosporine A. Representative FACS plots of mock-treated, WT EBV and ZKO EBV infected PBMCs on the day 3, 5, and 7 after infection and pre-gated on CD19^+^ B cell populations of live CD3^neg^ cells. The experiment was done in duplicates. Similar results were obtained for two donors.
(TIF)

Figure S2 **ZEBRA^+^ and gp350 cells in the spleen sections derived from WT and ZKO EBV infected animals six weeks post-infection.** Spleen sections were screened for presence of ZERBA^+^ (upper row) or gp350^+^ (lower row) cells at ×400 magnification and the total spleen area was defined at ×40 magnification. Data is composed of three independent experiments and represent mean ± SEM. p<0.01 by Wilcoxon signed rank test.
(TIF)

Figure S3 **Detection of EBV-specific CD8^+^ T cells in humanized NSG-A2tg mice six weeks after infection with WT and ZKO EBV.** Representative flow cytometry plots demonstrating staining of peripheral blood mononuclear cells (**A**) and splenocytes (**B**) using HLA-A*02 dextramers complexed with lytic (BMLF1, BRLF1), latent (LMP1, LMP2) EBV and control HIV gag antigen derived peptides. Pre-gated on the population of live human CD45^+^ CD3^+^ lymphocytes.
(PDF)

Figure S4 **Quantification of EBNA2-expressing B cells in spleen sections derived from WT and ZKO EBV infected animals six weeks post-infection.** (**A**) EBNA2-expressing cells were quantified in splenic sections for three independent experiments (n.s. p = 0.42). (**B**) Percentage of EBNA2-expressing B cells for WT and ZKO EBV infected animals in spleen sections was normalized to B cell numbers for three independent experiments (n.s. p = 0.28). Data represent mean ± SEM.
(TIF)

Figure S5 **NK and myeloid cell composition in the spleens and livers of WT and ZKO infected mice 6 weeks after infection.** (**A**) Quantification of cells expressing NKp46 (n.s. p _WT vs ZKO_ = 0.44), human CD68 (n.s. p_ WT vs ZKO_ = 0.13) and neutrophil elastase (n.s. p_ WT vs ZKO_ = 0.72) in the spleen sections from WT and ZKO EBV infected mice. (**B**) Quantification of cells expressing NKp46 (n.s. p_ WT:control vs tumor_ = 0.25), human CD68 (p _WT:control vs tumor_<0.05) and neutrophil elastase (n.s. p_ WT:control vs tumor_ = 0.11) in the hepatic tumors and control liver tissue in WT and ZKO EBV infected mice. (**C**) Percentages of CD3^neg^ CD4^neg^ CD19^neg^ lymphocytes (n.s. p = 0.90) and CD3^neg^ CD8^neg^ CD19^neg^ (n.s. p = 0.48) leucocytes, but not lymphocytes within human CD45^+^ cell population in the spleens of WT and ZKO EBV infected huNSG-A2tg mice. Data represent composite data from three independent experiments as mean ± SEM.
(PDF)

Figure S6 **Expression of activation and homing markers by LMP2- and BMLF1-specific CD8^+^ T cell clones.** Flow cytometry plots demonstrate activation and homing markers for an isotype control and two pairs of BMLF1-specific and LMP2-specific CD8^+^ T cell clones used for adoptive transfer into humanized mice. Pre-gated on the population of live human CD45^+^ CD3^+^ CD8^+^ cells.
(TIF)

Table S1 **T cell receptor variable gene usage by LMP2- and BMLF1-specific CD8^+^ T cell clones.**
(DOCX)

Table S2 **Overview of adoptive transfer experiments.**
(DOCX)

Table S3 The identification numbers of EBV complete wild type genome (strain B95.8), proteins and HLA-A*02-restricted epitopes used in the study.
(DOCX)

## References

[1] Young LS, Rickinson AB (2004) Epstein-Barr virus: 40 years on. Nat Rev Cancer 4: 757–768. 15510157 10.1038/nrc1452

[2] Kutok JL, Wang F (2006) Spectrum of Epstein-Barr virus-associated diseases. Annu Rev Pathol 1: 375–404. 18039120 10.1146/annurev.pathol.1.110304.100209

[3] Miller G, El-Guindy A, Countryman J, Ye J, Gradoville L (2007) Lytic cycle switches of oncogenic human gammaherpesviruses. Adv Cancer Res 97: 81–109. 17419942 10.1016/S0065-230X(06)97004-3

[4] Hislop AD, Taylor GS, Sauce D, Rickinson AB (2007) Cellular responses to viral infection in humans: lessons from epstein-barr virus. Annu Rev Immunol 25: 587–617. 17378764 10.1146/annurev.immunol.25.022106.141553

[5] Münz C, Bickham KL, Subklewe M, Tsang ML, Chahroudi A, et al (2000) Human CD4^+^ T lymphocytes consistently respond to the latent Epstein-Barr virus nuclear antigen EBNA1. J Exp Med 191: 1649–1660. 10811859 10.1084/jem.191.10.1649 PMC2193162

[6] Murray RJ, Kurilla MG, Brooks JM, Thomas WA, Rowe M, et al (1992) Identification of target antigens for the human cytotoxic T cell response to Epstein-Barr virus (EBV): implications for the immune control of EBV-positive malignancies. J Exp Med 176: 157–168. 1319456 10.1084/jem.176.1.157 PMC2119296

[7] Adhikary D, Behrends U, Moosmann A, Witter K, Bornkamm GW, et al (2006) Control of Epstein-Barr virus infection in vitro by T helper cells specific for virion glycoproteins. J Exp Med 203: 995–1006. 16549597 10.1084/jem.20051287 PMC2118290

[8] Steven NM, Annels NE, Kumar A, Leese AM, Kurilla MG, et al (1997) Immediate early and early lytic cycle proteins are frequent targets of the Epstein-Barr virus-induced cytotoxic T cell response. J Exp Med 185: 1605–1617. 9151898 10.1084/jem.185.9.1605 PMC2196300

[9] Cho YG, Gordadze AV, Ling PD, Wang F (1999) Evolution of two types of rhesus lymphocryptovirus similar to type 1 and type 2 Epstein-Barr virus. J Virol 73: 9206–9212. 10516028 10.1128/jvi.73.11.9206-9212.1999 PMC112954

[10] Leung C, Chijioke O, Gujer C, Chatterjee B, Antsiferova O, et al (2013) Infectious diseases in humanized mice. Eur J Immunol 43: 2246–2254. 23913412 10.1002/eji.201343815

[11] Ohashi M, Fogg MH, Orlova N, Quink C, Wang F (2012) An Epstein-Barr virus encoded inhibitor of Colony Stimulating Factor-1 signaling is an important determinant for acute and persistent EBV infection. PLoS Pathog 8: e1003095. 23300447 10.1371/journal.ppat.1003095 PMC3531511

[12] Marr-Belvin AK, Carville AK, Fahey MA, Boisvert K, Klumpp SA, et al (2008) Rhesus lymphocryptovirus type 1-associated B-cell nasal lymphoma in SIV-infected rhesus macaques. Vet Pathol 45: 914–921. 18984796 10.1354/vp.45-6-914 PMC2735115

[13] Strowig T, Gurer C, Ploss A, Liu YF, Arrey F, et al (2009) Priming of protective T cell responses against virus-induced tumors in mice with human immune system components. J Exp Med 206: 1423–1434. 19487422 10.1084/jem.20081720 PMC2715061

[14] Chijioke O, Müller A, Feederle R, Barros MH, Krieg C, et al (2013) Natural killer cells prevent infectious mononucleosis features by targeting lytic Epstein-Barr virus infection. Cell Rep 5: 1489–1498. 24360958 10.1016/j.celrep.2013.11.041 PMC3895765

[15] Strowig T, Chijioke O, Carrega P, Arrey F, Meixlsperger S, et al (2010) Human NK cells of mice with reconstituted human immune system components require preactivation to acquire functional competence. Blood 116: 4158–4167. 20671122 10.1182/blood-2010-02-270678 PMC2993621

[16] Gurer C, Strowig T, Brilot F, Pack M, Trumpfheller C, et al (2008) Targeting the nuclear antigen 1 of Epstein Barr virus to the human endocytic receptor DEC-205 stimulates protective T-cell responses. Blood 112: 1231–1239. 18519810 10.1182/blood-2008-03-148072 PMC2515117

[17] Meixlsperger S, Leung CS, Ramer PC, Pack M, Vanoaica LD, et al (2013) CD141^+^ dendritic cells produce prominent amounts of IFN-alpha after dsRNA recognition and can be targeted via DEC-205 in humanized mice. Blood 121: 5034–5044. 23482932 10.1182/blood-2012-12-473413 PMC3689250

[18] White RE, Ramer PC, Naresh KN, Meixlsperger S, Pinaud L, et al (2012) EBNA3B-deficient EBV promotes B cell lymphomagenesis in humanized mice and is found in human tumors. J Clin Invest 122: 1487–1502. 22406538 10.1172/JCI58092 PMC3314448

[19] Tsai MH, Raykova A, Klinke O, Bernhardt K, Gartner K, et al (2013) Spontaneous Lytic Replication and Epitheliotropism Define an Epstein-Barr Virus Strain Found in Carcinomas. Cell Rep 5: 458–70. 24120866 10.1016/j.celrep.2013.09.012

[20] Berger C, Day P, Meier G, Zingg W, Bossart W, et al (2001) Dynamics of Epstein-Barr virus DNA levels in serum during EBV-associated disease. J Med Virol 64: 505–512. 11468736

[21] Fonteneau JF, Larsson M, Somersan S, Sanders C, Münz C, et al (2001) Generation of high quantities of viral and tumor-specific human CD4^+^ and CD8^+^ T-cell clones using peptide pulsed mature dendritic cells. J Immunol Methods 258: 111–126. 11684128 10.1016/s0022-1759(01)00477-x

[22] Yousef S, Planas R, Chakroun K, Hoffmeister-Ullerich S, Binder TM, et al (2012) TCR bias and HLA cross-restriction are strategies of human brain-infiltrating JC virus-specific CD4^+^ T cells during viral infection. J Immunol 189: 3618–3630. 22942431 10.4049/jimmunol.1201612

[23] Ressing ME, Keating SE, van Leeuwen D, Koppers-Lalic D, Pappworth IY, et al (2005) Impaired transporter associated with antigen processing-dependent peptide transport during productive EBV infection. J Immunol 174: 6829–6838. 15905524 10.4049/jimmunol.174.11.6829

[24] Feederle R, Kost M, Baumann M, Janz A, Drouet E, et al (2000) The Epstein-Barr virus lytic program is controlled by the co-operative functions of two transactivators. Embo J 19: 3080–3089. 10856251 10.1093/emboj/19.12.3080 PMC203345

[25] Katsumura KR, Maruo S, Wu Y, Kanda T, Takada K (2009) Quantitative evaluation of the role of Epstein-Barr virus immediate-early protein BZLF1 in B-cell transformation. J Gen Virol 90: 2331–2341. 19553389 10.1099/vir.0.012831-0

[26] Hong GK, Gulley ML, Feng WH, Delecluse HJ, Holley-Guthrie E, et al (2005) Epstein-Barr virus lytic infection contributes to lymphoproliferative disease in a SCID mouse model. J Virol 79: 13993–14003. 16254335 10.1128/JVI.79.22.13993-14003.2005 PMC1280209

[27] Shultz LD, Saito Y, Najima Y, Tanaka S, Ochi T, et al (2010) Generation of functional human T-cell subsets with HLA-restricted immune responses in HLA class I expressing NOD/SCID/IL2r gamma^null^ humanized mice. Proc Natl Acad Sci U S A 107: 13022–13027. 20615947 10.1073/pnas.1000475107 PMC2919921

[28] Ma SD, Hegde S, Young KH, Sullivan R, Rajesh D, et al (2011) A new model of Epstein-Barr virus infection reveals an important role for early lytic viral protein expression in the development of lymphomas. J Virol 85: 165–177. 20980506 10.1128/JVI.01512-10 PMC3014199

[29] Melenhorst JJ, Leen AM, Bollard CM, Quigley MF, Price DA, et al (2010) Allogeneic virus-specific T cells with HLA alloreactivity do not produce GVHD in human subjects. Blood 116: 4700–4702. 20709906 10.1182/blood-2010-06-289991 PMC2996125

[30] Leen AM, Bollard CM, Mendizabal AM, Shpall EJ, Szabolcs P, et al (2013) Multicenter study of banked third-party virus-specific T cells to treat severe viral infections after hematopoietic stem cell transplantation. Blood 121: 5113–5123. 23610374 10.1182/blood-2013-02-486324 PMC3695359

[31] Luzuriaga K, Sullivan JL (2010) Infectious mononucleosis. N Engl J Med 362: 1993–2000. 20505178 10.1056/NEJMcp1001116

[32] Callan MF, Tan L, Annels N, Ogg GS, Wilson JD, et al (1998) Direct visualization of antigen-specific CD8^+^ T cells during the primary immune response to Epstein-Barr virus In vivo. J Exp Med 187: 1395–1402. 9565632 10.1084/jem.187.9.1395 PMC2212279

[33] Abbott RJ, Quinn LL, Leese AM, Scholes HM, Pachnio A, et al (2013) CD8^+^ T cell responses to lytic EBV infection: late antigen specificities as subdominant components of the total response. J Immunol 191: 5398–5409. 24146041 10.4049/jimmunol.1301629 PMC5580796

[34] Pudney VA, Leese AM, Rickinson AB, Hislop AD (2005) CD8^+^ immunodominance among Epstein-Barr virus lytic cycle antigens directly reflects the efficiency of antigen presentation in lytically infected cells. J Exp Med 201: 349–360. 15684323 10.1084/jem.20041542 PMC2213038

[35] Rooney CM, Smith CA, Ng CY, Loftin S, Li C, et al (1995) Use of gene-modified virus-specific T lymphocytes to control Epstein-Barr-virus-related lymphoproliferation. Lancet 345: 9–13. 7799740 10.1016/s0140-6736(95)91150-2

[36] Adhikary D, Behrends U, Boerschmann H, Pfunder A, Burdach S, et al (2007) Immunodominance of lytic cycle antigens in Epstein-Barr virus-specific CD4^+^ T cell preparations for therapy. PLoS ONE 2: e583. 17611619 10.1371/journal.pone.0000583 PMC1894652

[37] Haque T, Wilkie GM, Taylor C, Amlot PL, Murad P, et al (2002) Treatment of Epstein-Barr-virus-positive post-transplantation lymphoproliferative disease with partly HLA-matched allogeneic cytotoxic T cells. Lancet 360: 436–442. 12241714 10.1016/S0140-6736(02)09672-1

[38] Haque T, Wilkie GM, Jones MM, Higgins CD, Urquhart G, et al (2007) Allogeneic cytotoxic T-cell therapy for EBV-positive posttransplantation lymphoproliferative disease: results of a phase 2 multicenter clinical trial. Blood 110: 1123–1131. 17468341 10.1182/blood-2006-12-063008

[39] Doubrovina E, Oflaz-Sozmen B, Prockop SE, Kernan NA, Abramson S, et al (2012) Adoptive immunotherapy with unselected or EBV-specific T cells for biopsy-proven EBV^+^ lymphomas after allogeneic hematopoietic cell transplantation. Blood 119: 2644–2656. 22138512 10.1182/blood-2011-08-371971 PMC3311278

[40] Bolton DL, Minang JT, Trivett MT, Song K, Tuscher JJ, et al (2010) Trafficking, persistence, and activation state of adoptively transferred allogeneic and autologous Simian Immunodeficiency Virus-specific CD8^+^ T cell clones during acute and chronic infection of rhesus macaques. J Immunol 184: 303–314. 19949089 10.4049/jimmunol.0902413 PMC2797565

[41] Sokal EM, Hoppenbrouwers K, Vandermeulen C, Moutschen M, Leonard P, et al (2007) Recombinant gp350 vaccine for infectious mononucleosis: a phase 2, randomized, double-blind, placebo-controlled trial to evaluate the safety, immunogenicity, and efficacy of an Epstein-Barr virus vaccine in healthy young adults. J Infect Dis 196: 1749–1753. 18190254 10.1086/523813

[42] Cui X, Cao Z, Sen G, Chattopadhyay G, Fuller DH, et al (2013) A novel tetrameric gp350 1–470 as a potential Epstein-Barr virus vaccine. Vaccine 31: 3039–3045. 23665339 10.1016/j.vaccine.2013.04.071 PMC3700395

[43] Ogembo JG, Kannan L, Ghiran I, Nicholson-Weller A, Finberg RW, et al (2013) Human complement receptor type 1/CD35 is an Epstein-Barr Virus receptor. Cell Rep 3: 371–385. 23416052 10.1016/j.celrep.2013.01.023 PMC3633082

[44] Hui EP, Taylor GS, Jia H, Ma BB, Chan SL, et al (2013) Phase I trial of recombinant modified vaccinia ankara encoding Epstein-Barr viral tumor antigens in nasopharyngeal carcinoma patients. Cancer Res 73: 1676–1688. 23348421 10.1158/0008-5472.CAN-12-2448 PMC6485495

[45] Taylor GS, Haigh TA, Gudgeon NH, Phelps RJ, Lee SP, et al (2004) Dual stimulation of Epstein-Barr Virus (EBV)-specific CD4^+^- and CD8^+^-T-cell responses by a chimeric antigen construct: potential therapeutic vaccine for EBV-positive nasopharyngeal carcinoma. J Virol 78: 768–778. 14694109 10.1128/JVI.78.2.768-778.2004 PMC368843

[46] Ruiss R, Jochum S, Wanner G, Reisbach G, Hammerschmidt W, et al (2011) A virus-like particle-based Epstein-Barr virus vaccine. J Virol 85: 13105–13113. 21994444 10.1128/JVI.05598-11 PMC3233152

[47] Feederle R, Shannon-Lowe C, Baldwin G, Delecluse HJ (2005) Defective infectious particles and rare packaged genomes produced by cells carrying terminal-repeat-negative Epstein-Barr virus. J Virol 79: 7641–7647. 15919916 10.1128/JVI.79.12.7641-7647.2005 PMC1143645

[48] Adhikary D, Behrends U, Feederle R, Delecluse HJ, Mautner J (2008) Standardized and highly efficient expansion of Epstein-Barr virus-specific CD4^+^ T cells by using virus-like particles. J Virol 82: 3903–3911. 18272580 10.1128/JVI.02227-07 PMC2293016

[49] Pavlova S, Feederle R, Gartner K, Fuchs W, Granzow H, et al (2013) An Epstein-Barr virus mutant produces immunogenic defective particles devoid of viral DNA. J Virol 87: 2011–2022. 23236073 10.1128/JVI.02533-12 PMC3571473

[50] Leung CS, Maurer MA, Meixlsperger S, Lippmann A, Cheong C, et al (2013) Robust T cell stimulation by Epstein-Barr virus-transformed B cells after antigen targeting to DEC-205. Blood 121: 1584–94. 23297134 10.1182/blood-2012-08-450775 PMC3587321

