# Supplementary figures and images for: Adoptive Transfer of EBV Specific CD8+ T Cell Clones Can Transiently Control EBV Infection in Humanized Mice
Source: PLoS Pathog. 2014 Aug 28;10(8):e1004333. doi: 10.1371/journal.ppat.1004333 (PMC4148450; doi:10.1371/journal.ppat.1004333)

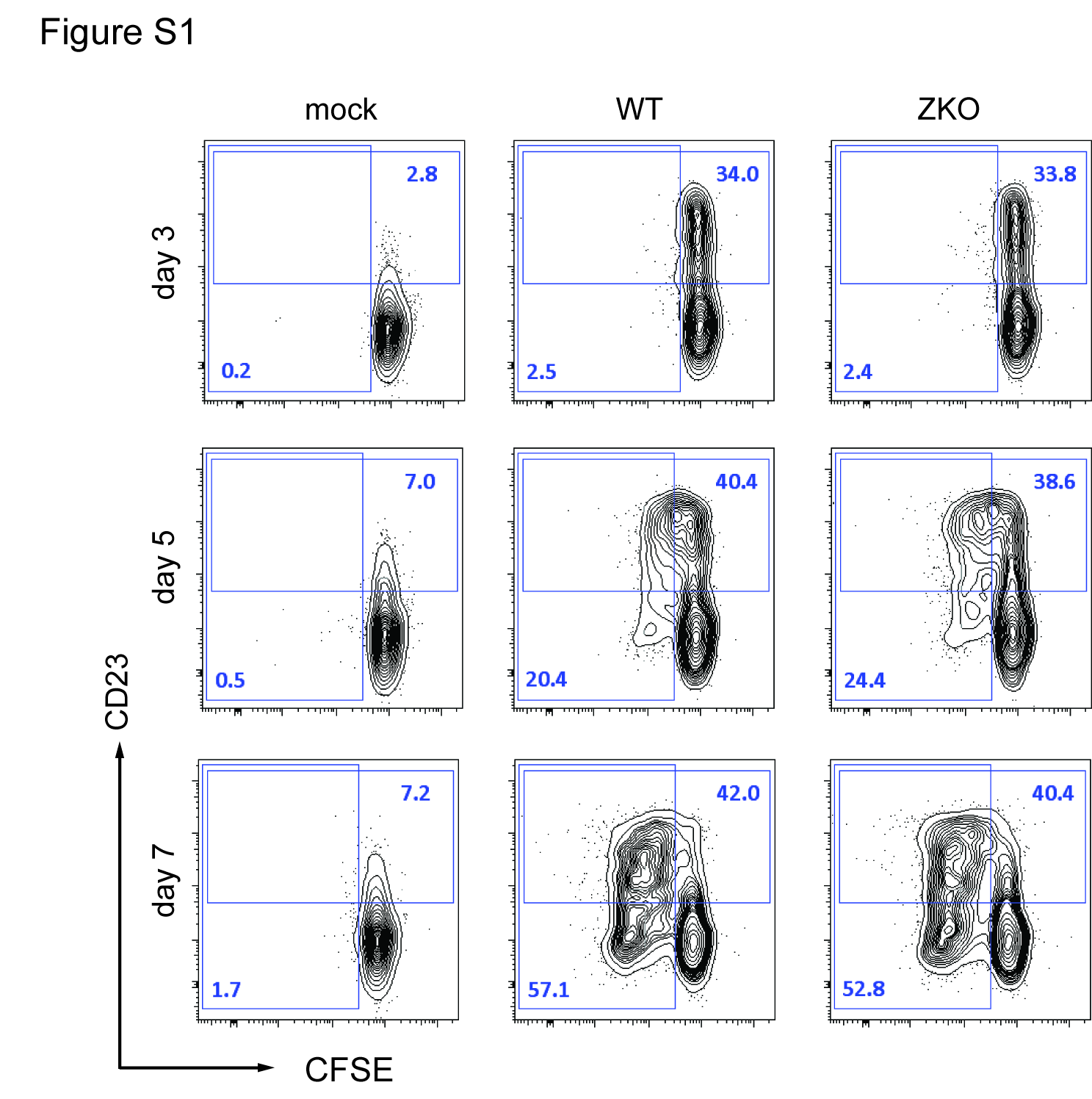

Supplement: Figure S1 — ZKO EBV deficient in lytic replication leads to up-regulation of B cell activation marker CD23 and subsequent proliferation. Bulk PBMCs were inoculated with WT EBV, ZKO EBV or mock treated in the presence of cyclosporine A. Representative FACS plots of mock-treated, WT EBV and ZKO EBV infected PBMCs on the day 3, 5, and 7 after infection and pre-gated on CD19+ B cell populations of live CD3neg cells. The experiment was done in duplicates. Similar results were obtained for two donors. (TIF) [file ppat.1004333.s001.tif]

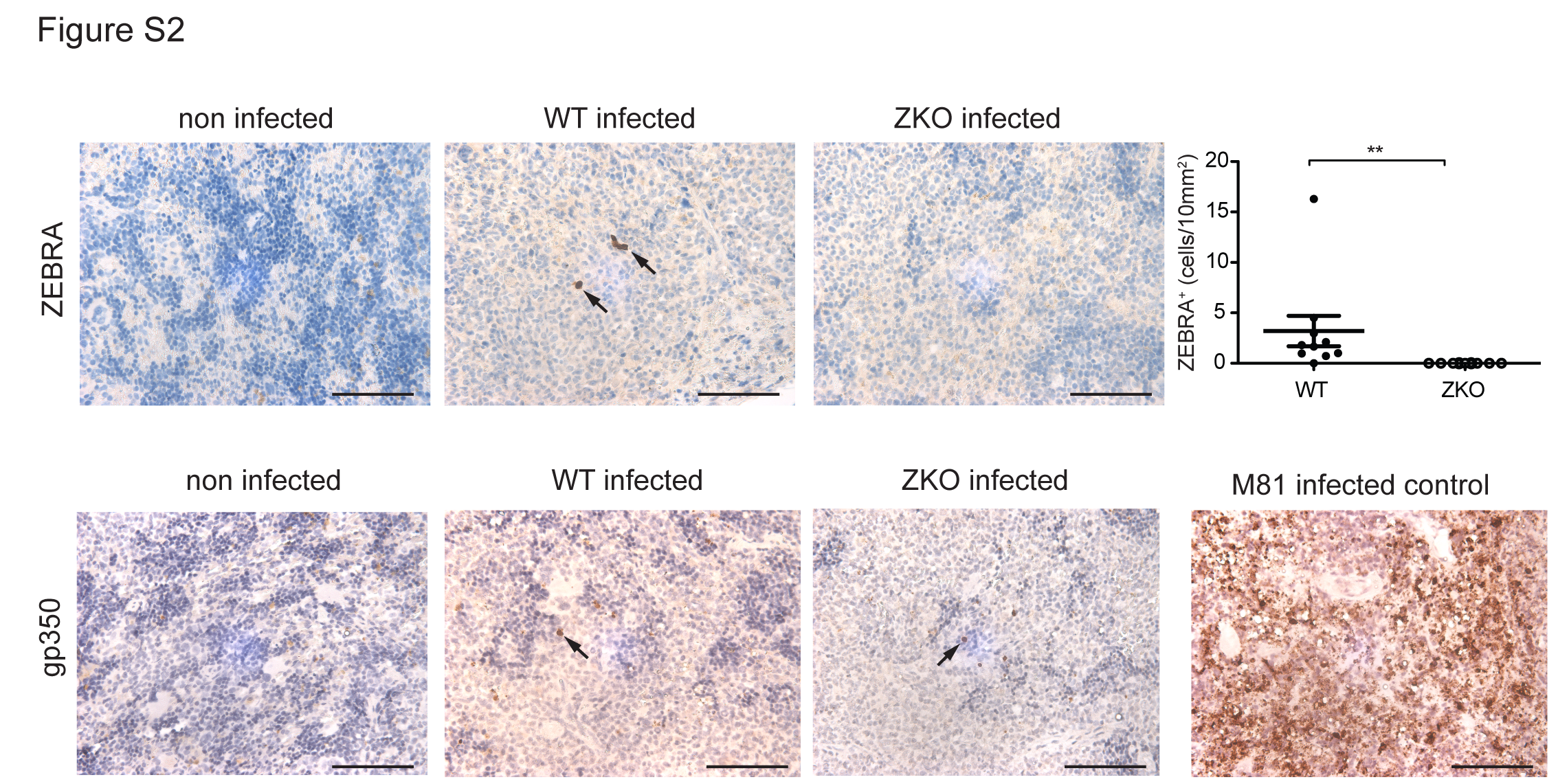

Supplement: Figure S2 — ZEBRA+ and gp350 cells in the spleen sections derived from WT and ZKO EBV infected animals six weeks post-infection. Spleen sections were screened for presence of ZERBA+ (upper row) or gp350+ (lower row) cells at ×400 magnification and the total spleen area was defined at ×40 magnification. Data is composed of three independent experiments and represent mean ± SEM. p<0.01 by Wilcoxon signed rank test. (TIF) [file ppat.1004333.s002.tif]

Figure S3

A

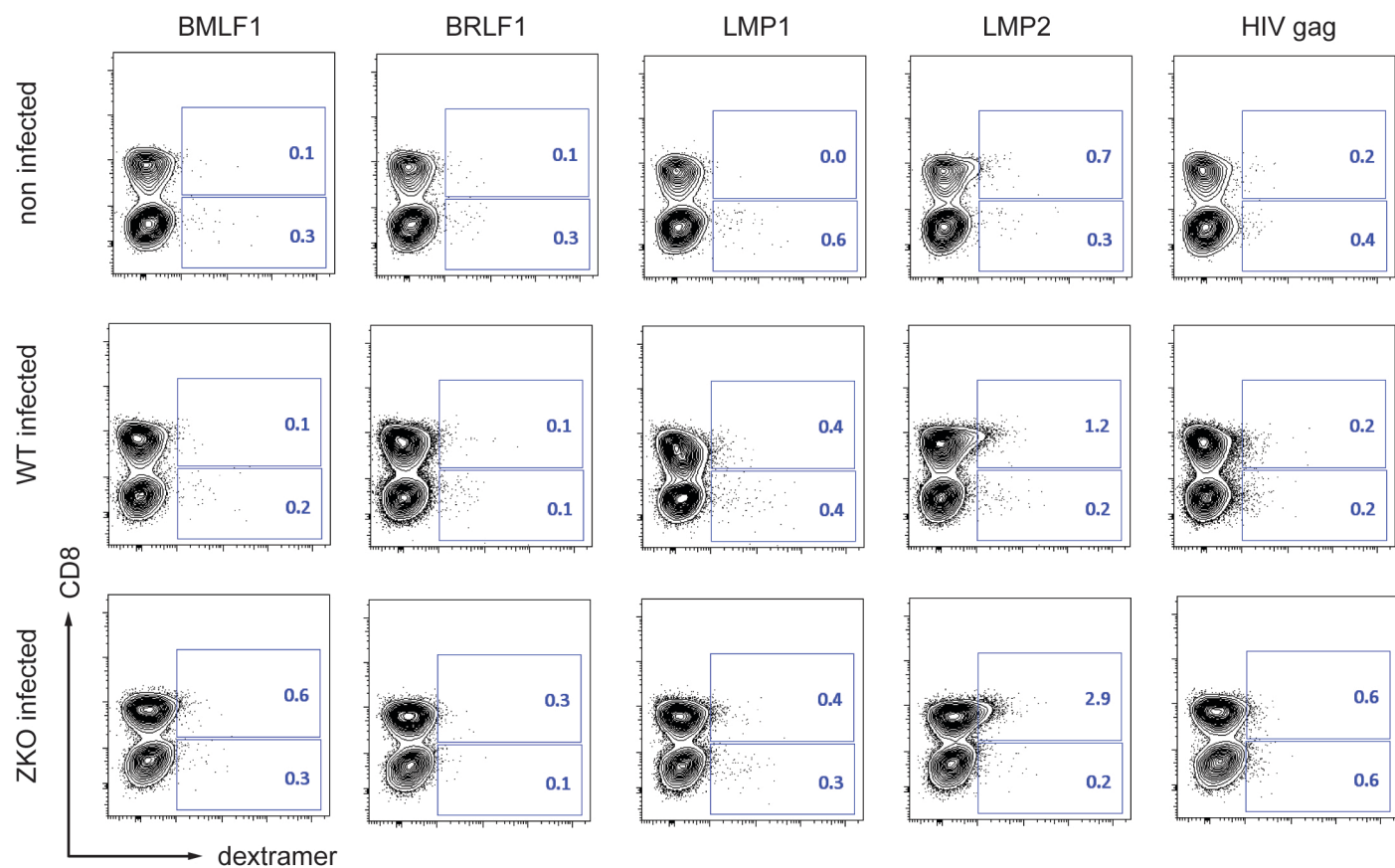

B

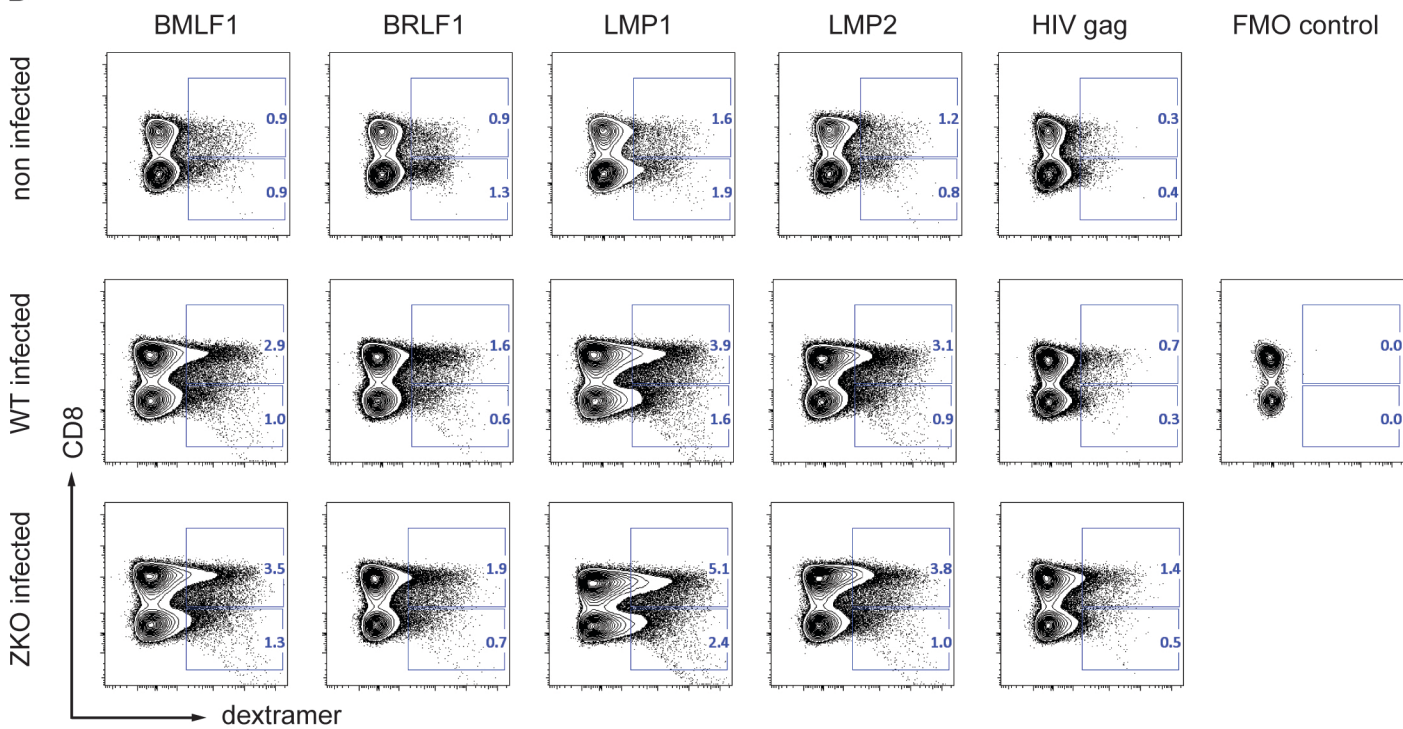

Supplement: Figure S3 — Detection of EBV-specific CD8+ T cells in humanized NSG-A2tg mice six weeks after infection with WT and ZKO EBV. Representative flow cytometry plots demonstrating staining of peripheral blood mononuclear cells (A) and splenocytes (B) using HLA-A*02 dextramers complexed with lytic (BMLF1, BRLF1), latent (LMP1, LMP2) EBV and control HIV gag antigen derived peptides. Pre-gated on the population of live human CD45+ CD3+ lymphocytes. (PDF) [file ppat.1004333.s003.pdf]

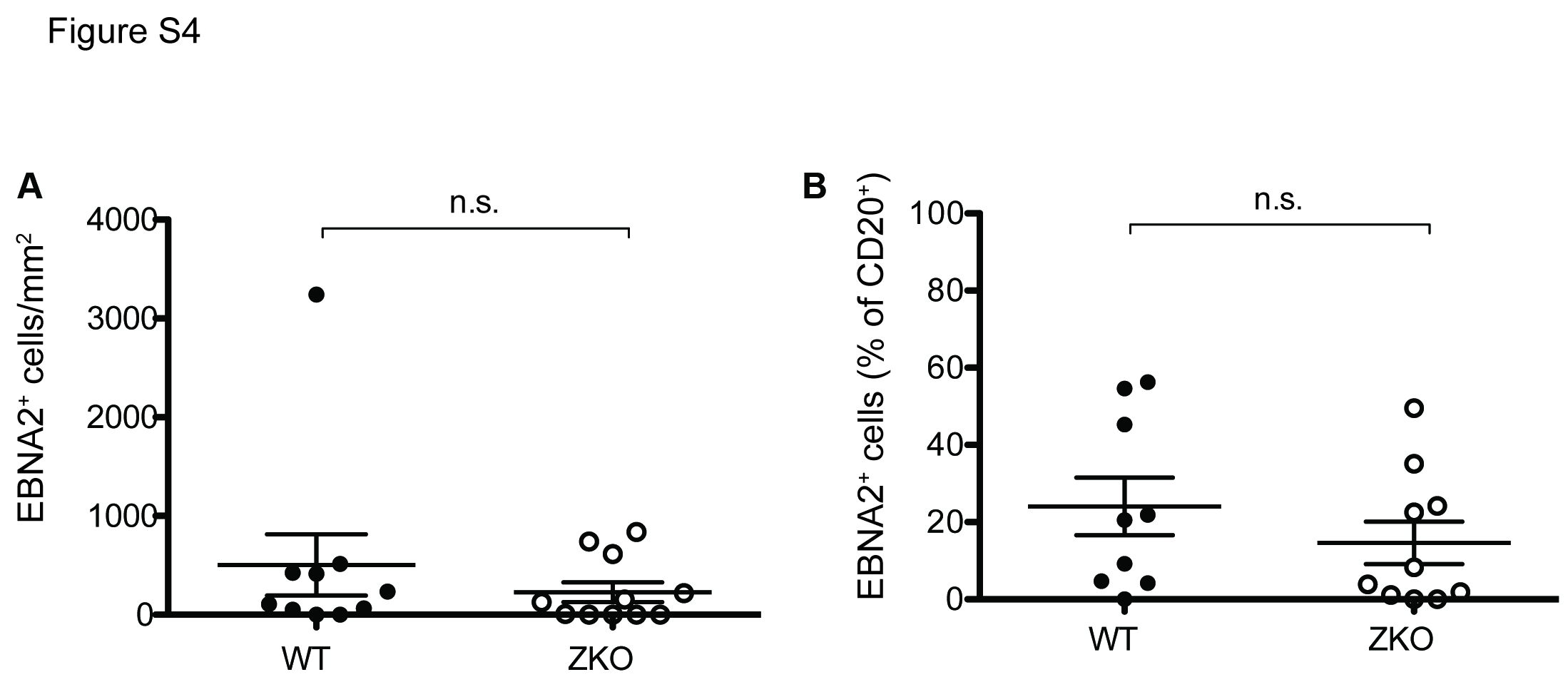

Supplement: Figure S4 — Quantification of EBNA2-expressing B cells in spleen sections derived from WT and ZKO EBV infected animals six weeks post-infection. (A) EBNA2-expressing cells were quantified in splenic sections for three independent experiments (n.s. p = 0.42). (B) Percentage of EBNA2-expressing B cells for WT and ZKO EBV infected animals in spleen sections was normalized to B cell numbers for three independent experiments (n.s. p = 0.28). Data represent mean ± SEM. (TIF) [file ppat.1004333.s004.tif]

Figure S5

A

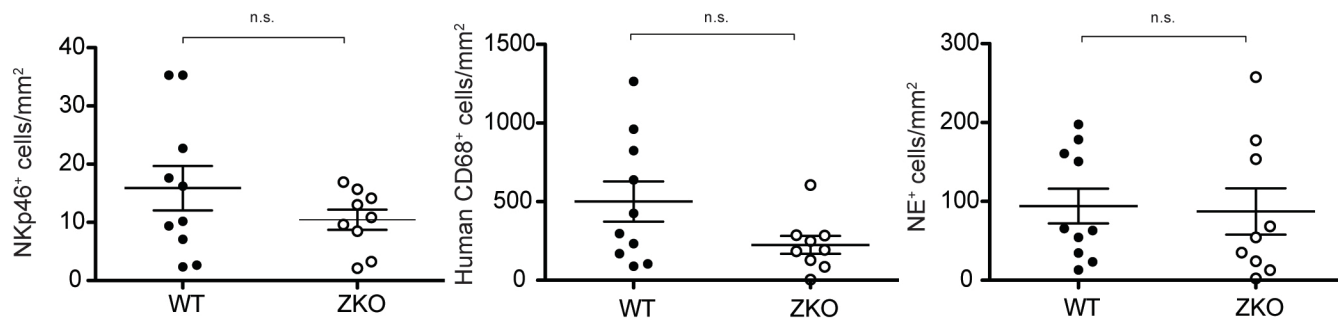

B

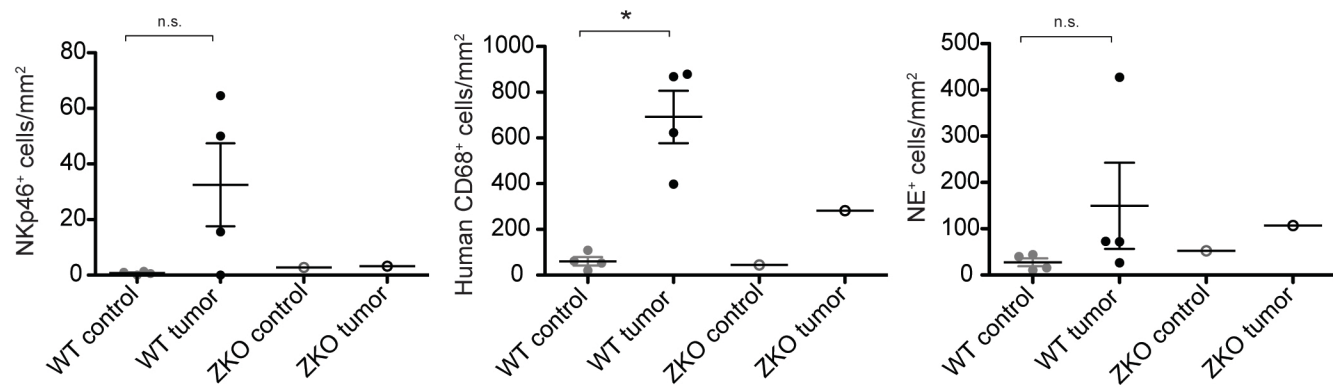

C

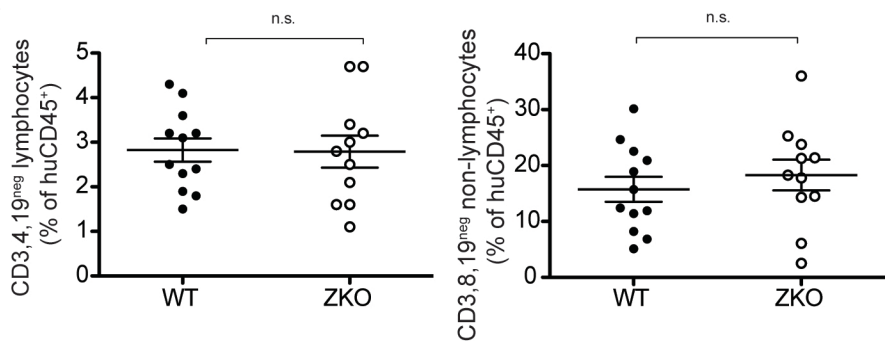

Supplement: Figure S5 — NK and myeloid cell composition in the spleens and livers of WT and ZKO infected mice 6 weeks after infection. (A) Quantification of cells expressing NKp46 (n.s. p WT vs ZKO = 0.44), human CD68 (n.s. p WT vs ZKO = 0.13) and neutrophil elastase (n.s. p WT vs ZKO = 0.72) in the spleen sections from WT and ZKO EBV infected mice. (B) Quantification of cells expressing NKp46 (n.s. p WT:control vs tumor = 0.25), human CD68 (p WT:control vs tumor<0.05) and neutrophil elastase (n.s. p WT:control vs tumor = 0.11) in the hepatic tumors and control liver tissue in WT and ZKO EBV infected mice. (C) Percentages of CD3neg CD4neg CD19neg lymphocytes (n.s. p = 0.90) and CD3neg CD8neg CD19neg (n.s. p = 0.48) leucocytes, but not lymphocytes within human CD45+ cell population in the spleens of WT and ZKO EBV infected huNSG-A2tg mice. Data represent composite data from three independent experiments as mean ± SEM. (PDF) [file ppat.1004333.s005.pdf]

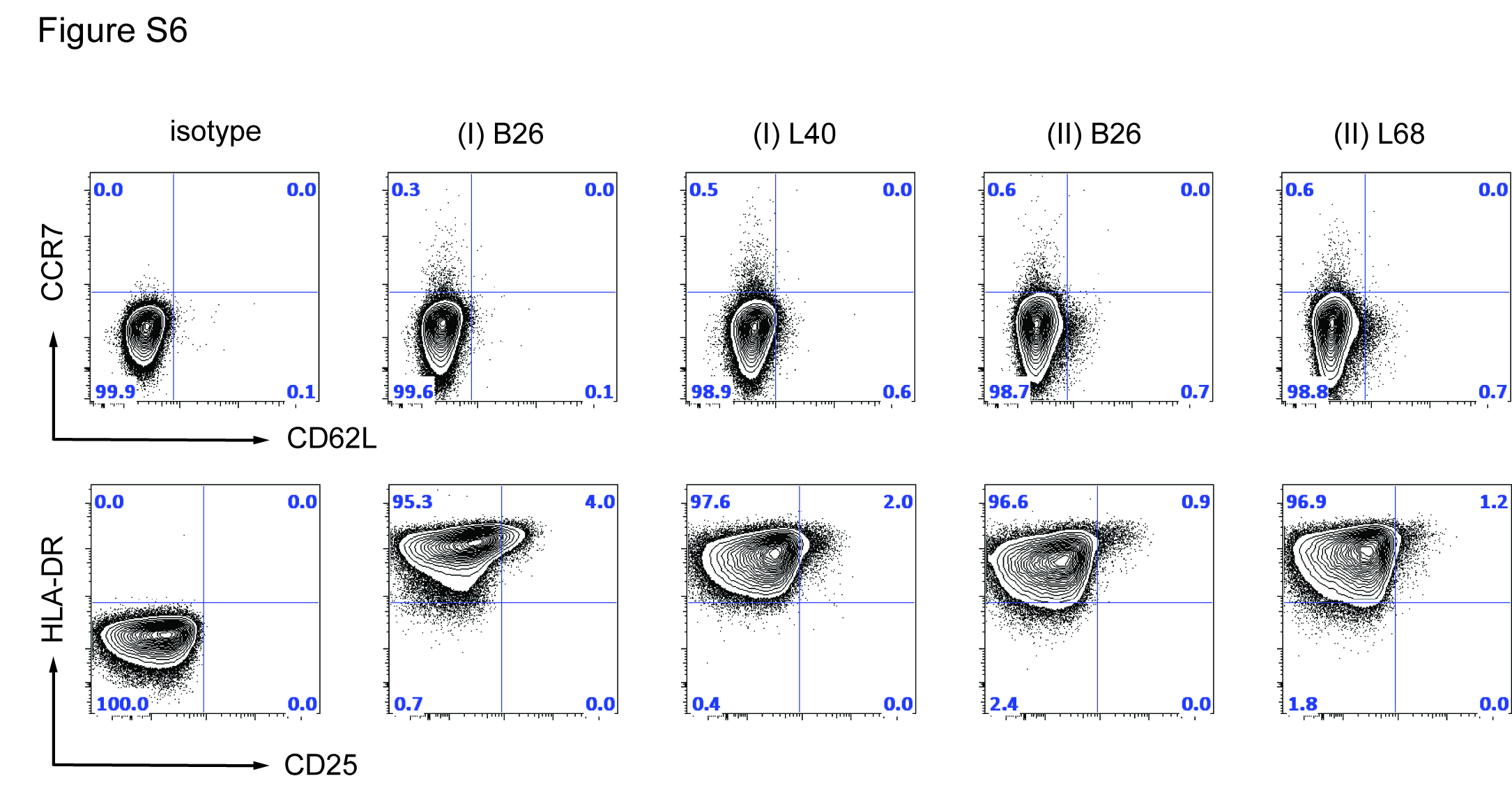

Supplement: Figure S6 — Expression of activation and homing markers by LMP2- and BMLF1-specific CD8+ T cell clones. Flow cytometry plots demonstrate activation and homing markers for an isotype control and two pairs of BMLF1-specific and LMP2-specific CD8+ T cell clones used for adoptive transfer into humanized mice. Pre-gated on the population of live human CD45+ CD3+ CD8+ cells. (TIF) [file ppat.1004333.s006.tif]
