# Supplementary material for: Adoptive Transfer of EBV Specific CD8+ T Cell Clones Can Transiently Control EBV Infection in Humanized Mice
Source: PLoS Pathog. 2014 Aug 28;10(8):e1004333. doi: 10.1371/journal.ppat.1004333 (PMC4148450; doi:10.1371/journal.ppat.1004333)
Supplement: Table S1 — T cell receptor variable gene usage by LMP2- and BMLF1-specific CD8+ T cell clones. (DOCX) [file ppat.1004333.s007.docx]

Table S1. T cell receptor variable gene usage by LMP2- and BMLF1-specific CD8^+^ T cell clones.

| **TCC** | **CDR3b aa sequence** | **CDR3b nucleotide sequence** | **CDR3b length** | **TRBV locus** | **TRBJ locus** | **TRBD locus** |
| --- | --- | --- | --- | --- | --- | --- |
| B1 | CSARTGVGNTIYF | tgc agt gct agg acg ggg gtt gga aac acc ata tat ttt | 11 | TRBV 20-1 | TRBJ 1-3*01 | TRBD 2*01 |
| B26 | CSARDSTGNGYTF | tgc agt gct aga gat tcg aca ggg aat ggc tac acc ttc | 11 | TRBV 20-1 | TRBJ 1-2*01 | TRBD 1*01 |
| B30 | CASSRGTVAPGELFF | tgt gcc agc agt cgg ggg acg gtc gca ccg ggg gag ctg ttt ttt | 13 | TRBV 2 | TRBJ 2-2*01 | TRBD 2*01 |
| B61 | CSARDRVGNTIYF | tgc agt gct aga gat agg gtg gga aac acc ata tat ttt | 11 | TRBV 20-1 | TRBJ 1-3*01 | TRBD 1*01 |
| L58 | CASSYSFGGAPAQETQYF | tgt gcc agc agt tat tcc ttt ggg ggg gcc ccc gct caa gag acc cag tac ttc | 16 | TRBV 6-5*01 | TRBJ 2-5*01 | TRBD2*01 |
| L60 | CASSSEGQASSYEQYF | tgc gcc agc agc tcc gag gga cag gca agc tcc tac gag cag tac ttc | 14 | TRBV 5-1*01 | TRBJ 2-7*01 | TRBD 1*01 |
| L68 | CASSSEGQASSYEQYF | tgc gcc agc agc tcc gag gga cag gca agc tcc tac gag cag tac ttc | 14 | TRBV 5-1*01 | TRBJ 2-7*01 | TRBD 1*01 |
| **TCC** | **CDR3a aa sequence** | **CDR3a nucleotide sequence** | **CDR3a length** | **TRAV locus** | **TRAJ locus** | **-** |
| B26 | CAEDNNARLMF | tgt gca gag gat aac aat gcc aga ctc atg ttt | 9 | TRAV  5*01 | TRAJ  31*01 | - |
| B30 | CVVNGMDSSYKLXF | tgt gtg gtg aac ggg atg gat agc agc tat aaa ttg atn ttc | 12 | TRAV  12-1*01 | TRAJ  12*01 | - |
| L58 | CAASAQDDKIIF | tgt gca gca agc gcg caa gat gac aag atc atc ttt | 10 | TRAV  29 | TRAJ  30*01 | - |
| L60 | CACSGAGSYQLTF | tgt gca tgc tct ggg gct ggg agt tac caa ctc act ttc | 11 | TRAV  25*01 | TRAJ  28*01 | - |
| L68 | CACSGAGSYQLTF | tgt gca tgc tct ggg gct ggg agt tac caa ctc act ttc | 11 | TRAV  25*01 | TRAJ  28*01 | - |
