# Supplementary material for: Adoptive Transfer of EBV Specific CD8+ T Cell Clones Can Transiently Control EBV Infection in Humanized Mice
Source: PLoS Pathog. 2014 Aug 28;10(8):e1004333. doi: 10.1371/journal.ppat.1004333 (PMC4148450; doi:10.1371/journal.ppat.1004333)
Supplement: Table S2 — Overview of adoptive transfer experiments. (DOCX) [file ppat.1004333.s008.docx]

Table S2. Overview of adoptive transfer experiments.

|  | | |  | **Clone avidity [M]** ^(1)^ | |
| --- | --- | --- | --- | --- | --- |
| **Cohort N°** | **N of mice** | **Duration** | **Recipient genotype (MHC class I)** | **BMLF1-T** | **LMP2-T** |
| Cohort 1 | 17 | 4 weeks | A*02, A*11; B*35, B*44 | 2.02x10^-6^ | 1.35x10^-6^ |
| Cohort 2 | 22 | 6 weeks | A*02, A*11; B*40, B*46 | 5.06x10^-6^ | 3.47x10^-8^ |
| Cohort 3 | 18 | 4 weeks | A*02, A*23; B*27, B*49 | 4.83x10^-7^ | 4.44x10^-7^ |
| Cohort 4 | 17 | 4 weeks | A*01, A*02; B*13, B*44 | 5.50x10^-7^ | 3.96x10^-7^ |
| Cohort 5 | 20 | 4 weeks | A*01, A*02; B*13, B*44 | 1.07x10^-5^ | 2.70x10^-6^ |

Donor genotype (MHC class I): A*02, A*68, B*44, B*07

^(1)^ Clone avidity was defined as cognate peptide concentration [M] with 50% of optimal IFNγ secretion.
