# Supplementary material for: Adoptive Transfer of EBV Specific CD8+ T Cell Clones Can Transiently Control EBV Infection in Humanized Mice
Source: PLoS Pathog. 2014 Aug 28;10(8):e1004333. doi: 10.1371/journal.ppat.1004333 (PMC4148450; doi:10.1371/journal.ppat.1004333)
Supplement: Table S3 — The identification numbers of EBV complete wild type genome (strain B95.8), proteins and HLA-A*02-restricted epitopes used in the study. (DOCX) [file ppat.1004333.s009.docx]

Table S3. The identification numbers of EBV complete wild type genome (strain B95.8), proteins and HLA-A*02-restricted epitopes used in the study.

| Gene name  (GenBank ID AJ507799.2) | Protein | | | HLA-A*02-restricted epitope | |
| --- | --- | --- | --- | --- | --- |
|  | Names | UniProtKB/ Swiss-Prot ID | Length | Sequence (aa) | Immune Epitope Database ID |
| BZLF1 | Transactivator protein BZLF1, EB1, ZEBRA, Zta | P03206 | 245 aa | - | - |
| BMLF1 | BMLF1 protein, EB2, protein SM, Mta | Q04360 | 479 aa | GLCTLVAML  (259-267) | 20788 |
| LMP2 | Latent membrane  protein 2, terminal  protein | P13285 | 497 aa | CLGGLLTMV (426–434) | 6568 |
